# Neuromuscular and neuromechanical assessments of respiratory performance in the *mdx* mouse model of Duchenne muscular dystrophy across the natural history of disease

**DOI:** 10.1113/EP093392

**Published:** 2026-02-03

**Authors:** Michael N. Maxwell, Christopher G. Wilson, Mai K. Elmallah, Federica Trucco, Ken D. O'Halloran

**Affiliations:** ^1^ Department of Physiology University College Cork Cork Ireland; ^2^ Division of Physiology, Department of Basic Sciences Loma Linda University Loma Linda California USA; ^3^ Division of Pulmonary and Sleep Medicine, Department of Pediatrics Duke University Durham North Carolina USA; ^4^ Department of Neurosciences, Rehabilitation, Ophthalmology, Genetics, Maternal and Child Health University of Genova Genova Italy; ^5^ Paediatric Neurology and Muscular Diseases Unit IRCCS Istituto Giannina Gaslini Genova Italy

**Keywords:** Duchenne muscular dystrophy, *mdx* mouse, neural respiratory drive, neuromechanical efficiency, respiratory electromyogram spectrum parameters, tension–time index

## Abstract

Duchenne muscular dystrophy (DMD) is a severe life‐limiting X‐linked neuromuscular disorder characterised by progressive skeletal muscle degeneration and respiratory failure. The *mdx* mouse, lacking dystrophin, is the most widely used preclinical model of DMD, yet the trajectory of respiratory dysfunction in this model remains incompletely defined. We evaluated neural respiratory drive (NRD), neuromechanical efficiency (NME), tension–time index (TTI), inspiratory drive rate and electromyographic (EMG) frequency spectrum parameters in the diaphragm, external intercostal and parasternal muscles across the natural history of disease (aged 1–16 months). Despite early and persistent reductions in EMG activity and frequency spectrum parameters in *mdx* mice, NRD and TTI in respiratory muscles were largely equivalent to controls. NME was paradoxically increased in *mdx* mice, likely reflecting compensatory recruitment of accessory muscles rather than improved contractile efficiency of the major inspiratory muscles of breathing. The area under the pressure–time curve during sustained tracheal occlusion was reduced in *mdx* mice at 1 month of age but was equivalent to wild‐type values at all other ages, demonstrating robust compensation even in advanced disease. No significant differences in inspiratory duty cycle, respiratory muscle effort or TTI were observed across groups. We conclude that assessments of integrative respiratory morbidity in *mdx* mice should focus on animals aged ≥16 months or alternative models with accelerated disease progression. Our results underscore the need for refined translational models and highlight the importance of integrating EMG‐based indices for early detection and monitoring of respiratory compromise in DMD.

## Abstract (highlights)

- **What is the central question of this study?**
  Reliable temporal biomarkers of respiratory involvement in Duchenne muscular dystrophy (DMD) are needed: are clinically relevant indices of respiratory neuromuscular performance altered across the natural history of disease in the *mdx* mouse model of DMD?
- **What is the main finding and its importance?**
  Several established indices of neuromuscular and neuromechanical performance in *mdx* mice were equivalent to wild‐type controls even in advanced disease. Studies in *mdx* mice >16 months of age or logistically convenient models of rapid neuromuscular disease progression are required to facilitate the study of ventilatory insufficiency, providing a platform to assess the efficacy of existing and emerging therapies for DMD.

## 1 INTRODUCTION

Duchenne muscular dystrophy (DMD) is a life‐limiting X‐linked neuromuscular disease, caused by mutations of the *DMD* gene that encodes the protein dystrophin (Hoffman et al., 1987). Dystrophin is part of the transmembrane dystrophin glycoprotein complex that connects the F‐actin of the cytoskeleton to the extracellular matrix via the sarcolemma, providing stability to the muscle membrane (Gao & McNally, 2015). Dystrophin deficiency results in skeletal muscle damage, immune cell infiltration, chronic inflammation and muscle wasting (Deconinck & Dan, 2007; Duan et al., 2021; McDouall et al., 1990; Schmalbruch, 1984). In DMD, there is a progressive decline in respiratory muscle strength and pulmonary function culminating in ventilatory insufficiency and consequential cardiorespiratory failure (De Bruin et al., 1997; Eagle et al., 2002; Ishikawa et al., 2011; Khirani et al., 2014; Smith et al., 1989).

It is well documented that despite corticosteroid treatment and its early ambulatory and respiratory benefits, respiratory function declines at a steady rate annually after reaching peak capacity (Fauroux et al., 2015; Khirani et al., 2014). The pathophysiological mechanisms underlying respiratory involvement in DMD are still poorly understood hampering its treatment (Trucco & O'Halloran, 2025). Spirometry and respiratory muscle testing as currently employed in DMD to assess the stage of respiratory impairment are not sufficient to fully characterise the trajectory of respiratory compromise from onset to end‐stage disease (Finder, 2009; Finder et al., 2004; Hudson et al., 2025; Sheehan et al., 2018). Complementary recordings of respiratory pressures and electromyography (EMG) could potentially complement standard assessment in charting the natural history of DMD by evaluating temporal progression of the disease (Fauroux et al., 2009, 2015; Finder, 2009; Finder et al., 2004; Hahn et al., 1997). A comprehensive battery of reliable and reproducible respiratory muscle tests could facilitate a pathology‐based approach to the clinical management of respiratory morbidity in DMD and could provide more sensitive markers to document the efficacy and/or effectiveness of established and novel therapies.

Respiratory EMG recordings can be used to assess the pattern and intensity of muscle activation. It is recommended that routine monitoring of respiratory muscle function should be employed to guide respiratory management in DMD (Finder, 2009; Finder et al., 2004; Hudson et al., 2025). Respiratory EMG is not utilised clinically in DMD despite a joint consensus for normal respiratory testing by the European Respiratory Society and American Thoracic Society suggesting that respiratory EMG can be used to detect and diagnose neuromuscular pathology (ATS/ERS, 2002), and when coupled with tests of mechanical function, can be reliably used to assess the efficacy of contractile function (Finder, 2009; Finder et al., 2004; Hudson et al., 2025; McManus et al., 2020).

EMG captures and displays the intricate electrical activity produced by motor units as they regulate both voluntary and reflex muscle movements. EMG signals serve as a key diagnostic tool for various neuromuscular and neurological conditions, including myopathy and amyotrophic lateral sclerosis (Samanta et al., 2022). Neuromuscular disorders such as DMD alter the morphology and physiology of the neuromuscular junction which are critical to motor unit health (Lovering et al., 2020; Ng & Ljubicic, 2020). Variations in EMG signal amplitude and spectral characteristics can reveal and document the balance between central and peripheral fatigue mechanisms in neuromuscular disorders (McManus et al., 2017). Myopathic conditions including DMD often show reduced low‐frequency spectral content in EMG signals due to the presence of shorter‐duration, polyphasic motor unit potentials resulting from muscle fibre degeneration and motor unit remodelling (Frascarelli et al., 1988; Kumar Bhoi et al., 2013; Ng & Ljubicic, 2020; Pandeya et al., 2023).

One of the most widely studied models of DMD is the dystrophin deficient *mdx* mouse. Our research group has previously demonstrated compromised respiratory muscle form and function in the *mdx* mouse with pronounced structural remodelling, weakness and impaired respiratory control (Burns, Murphy et al., 2019; Mhandire et al., 2022; O'Halloran et al., 2023; Slyne et al., 2025). EMG activity of the obligatory muscles of breathing in the *mdx* mouse is reduced, which likely reflects impaired neuromuscular function pertinent to respiratory performance (Burns, Murphy et al., 2019; O'Halloran et al., 2023; Personius & Sawyer, 2006; Slyne et al., 2025). We have previously investigated inspiratory pressure and EMG activity in 1‐, 4‐, 8‐, 12‐ and 16‐month‐old wild‐type and *mdx* mice (O'Halloran et al., 2023). Despite profound diaphragm muscle dysfunction, peak inspiratory pressure is preserved over a protracted period of the natural history of disease. Our work suggests that compensation is afforded by accessory respiratory muscles, including abdominal muscles, facilitated by the remodelled stiffened dystrophic diaphragm, as distinct from enhanced facilitation of respiratory motor drive in accessory motor pathways (O'Halloran et al., 2023).

In the current study, we re‐analysed data from our previous study (O'Halloran et al., 2023) to assess several clinically relevant indices of respiratory neuromuscular performance including neural respiratory drive, neuromuscular efficiency, tension–time index, inspiratory drive rate, EMG frequency spectrum analyses, total power, measures of entropy and the pressure–time relationship during sustained peak activation in wild‐type and *mdx* mice at 1, 4, 8, 12 and 16 months of age. Our aim was to further characterise respiratory performance in the most widely used mouse model of DMD over the natural history of the disease. We hypothesised that clinically relevant indices of respiratory performance would be impaired in *mdx* mice especially in advanced disease, providing useful translational biomarkers of DMD progression and offering a platform for the assessment of established and novel therapeutics for respiratory compromise in DMD.

## 2 METHODS

### 2.1 Ethical approval

Procedures on live animals were performed under project authorisations (AE19130/P117 and AE19130/P157) from the Health Products Regulatory Authority in accordance with Irish and European law with prior ethical approval by University College Cork (AEEC 2019/013 and AEEC 2021/019). Experiments were carried out in accordance with guidelines and requirements laid down by University College Cork's Animal Welfare Body and conformed to the principles and regulations described by Grundy (2015). All data in the current study were derived from *de novo* retrospective analyses of data collected in our previously published study (O'Halloran et al., 2023).

### 2.2 Experimental animals

Breeding pairs for wild‐type (C57BL/10ScSnJ) and *mdx* (C57BL/10ScSn‐Dmd*mdx*/J) mice were purchased from The Jackson Laboratory (Bar Harbor, ME, USA) and colonies were established at University College Cork's specific pathogen‐free facility. Studies were performed in male mice. Animals were housed in individually ventilated cages in temperature‐ and humidity‐controlled rooms, operating on a 12 h light–12 h dark cycle with food and water available ad libitum.

### 2.3 Respiratory EMGs and inspiratory pressure recordings

In wild‐type and *mdx* mice, anaesthesia was induced with 5% isoflurane in 60% O_2_ (balance N_2_) in an induction chamber. Mice were subsequently placed in the supine position and received 2% isoflurane in 60% O_2_ (balance N_2_) by nose‐cone delivery. Mice were gradually transitioned from isoflurane to urethane anaesthesia (1.7 g kg^−1^ i.p. in total given in three injections) over a 25‐min period. Body temperature was maintained at 37°C via a rectal probe and thermostatically controlled heating blanket (Harvard Apparatus, Holliston, MA, USA). Adequacy of depth of anaesthesia to permit surgical procedures was determined by an absent pedal withdrawal reflex and cardiac and respiratory frequency response to noxious pinch. Supplemental anaesthetic was administered over the course of the experimental protocol to ensure adequacy of depth of anaesthesia determined by the absence of palpebral and withdrawal reflexes and whisking, and stability of cardiorespiratory recordings. The final cumulative dose of urethane administered was 2.1–2.5 g kg^−1^ i.p. A pulse oximeter clip (MouseOx, Starr Life Sciences Corp., Oakmount, PA, USA) was placed on a shaved thigh for the measurement of peripheral capillary O_2_ saturation ($S_{pO_{2}}$). A mid‐cervical tracheotomy was performed. All animals were maintained with a bias flow of supplemental O_2_ ($F_{iO_{2}}$ = 0.60) under baseline conditions. End‐tidal carbon dioxide (ETCO_2_) was measured from a side‐arm of the tracheal cannula (MicroCapStar, CWE, Ardmore, PA, USA). Oesophageal pressure was measured using a pressure‐tip catheter (Mikro‐Tip, Millar Inc., Houston, TX, USA), which was positioned in the thoracic oesophagus through the mouth. The catheter was advanced into the stomach to record positive pressure changes during inspiration and then withdrawn into the lower oesophagus where stable phasic sub‐atmospheric pressure swings during inspiration were observed. Concentric needle monopolar recording electrodes (26G; Natus Manufacturing Ltd, Gort, Ireland) were inserted into the middle costal region of the diaphragm on the right‐hand side for the continuous measurement of diaphragm EMG activity. In addition, concentric needle monopolar electrodes were inserted into an external intercostal (EIC), in the second to fourth rostro‐ventral intercostal space, for the measurement of EIC EMG and into the parasternal (PS), second or third space, for the measurement of PS EMG. EMG signals were amplified (×5000) (Neurolog system, Digitimer Ltd, Welwyn Garden City, UK), and band‐passed filtered (50–5000 Hz). All signals were passed through an analog‐to‐digital converter (Powerlab r8/30; ADInstruments, Colorado Springs, CO, USA) and were acquired using LabChart 8 (ADInstruments) sampled at 20 kHz (EMG) or 1 kHz (other parameters).

### 2.4 Experimental protocol

Following instrumentation, animals were allowed to stabilise before baseline parameters were measured. Next, a pneumotachometer was connected to the tracheal cannula to record respiratory airflow for a period of 1–2 min. The pneumotachometer and ETCO_2_ were disconnected, and following a baseline period, animals were challenged with a single sustained tracheal occlusion for up to 30–40 s until peak inspiratory efforts to task failure (decline in pressure generation from the sustained peak nadir during occlusion) were observed in the inspiratory pressure recordings during sustained maximum non‐ventilatory efforts. Following recovery, animals were again instrumented for the measurement of ETCO_2_ and tracheal airflow, and parameters were recorded during a newly established second baseline (pre‐vagotomy) period. Subsequently, the vagi were sectioned bilaterally at the cervical level. Respiratory parameters were recorded under steady‐state conditions for at least 10 min following vagotomy. Next, animals were challenged with hypercapnic hypoxia ($F_{iO_{2}}$ = 0.15/$F_{\mathrm{iC}O_{2}}$ = 0.06; 2 min) to examine the effects of chemostimulation on diaphragm, EIC and PS EMG activities and ventilatory parameters. Following the experimental protocol, anaesthetised mice were killed by cervical dislocation and death was confirmed by the absence of cardiac rhythm.

### 2.5 Data analysis

#### 2.5.1 Neural respiratory drive

Neural respiratory drive (NRD) is a physiological index of the proportional activation of a muscle relative to maximum, used to infer the mechanical load on the respiratory muscles (Cavalcante et al., 2024; Jolley et al., 2009; MacBean et al., 2016; Murphy et al., 2011; Onofri et al., 2018; Reilly et al., 2016, 2011; Steier et al., 2017). NRD is expressed as EMG of the diaphragm during tidal breathing as a percentage of peak EMG activity obtained during a maximum inspiratory manoeuvre. Breath‐by‐breath calculation of NRD can be measured in real time (Cavalcante et al., 2024; Jolley et al., 2009; MacBean et al., 2016; Murphy et al., 2011; Onofri et al., 2018; Reilly et al., 2016, 2011; Steier et al., 2017). NRD has been used as a biomarker of disease severity in COPD (Jolley et al., 2009; Murphy et al., 2011), obesity hypoventilation syndrome (Onofri et al., 2018), hypertension (Cavalcante et al., 2024) and cystic fibrosis (Reilly et al., 2013, 2011, 2016).

Respiratory EMG values were determined across all states (baseline, post‐vagotomy and subsequent chemostimulation) and represented as a percentage of the maximum values determined during five consecutive peak efforts during the single sustained tracheal occlusion challenge to obtain the NRD index (%) as shown in the equation below.

$$\mathrm{NRD}\;=\frac{\mathrm{EMG}}{\mathrm{EM}G_{\mathrm{MAX}}}\;\times 100$$

#### 2.5.2 Neuromechanical efficiency

The combination of inspiratory pressure and EMG provides a proxy measure for neuromechanical efficiency (NME) (also known as neuromechanical coupling). NME reflects the efficiency of converting neural activation to pressure generation (Bellani et al., 2013; Jansen et al., 2018; Lozano‐Garcia et al., 2019; Lozano‐García et al., 2021), reflecting the magnitude of inspiratory pressure that can be generated for each unit of diaphragm electrical activity (Bellani et al., 2013; Jansen et al., 2018). NME has been used to estimate breath‐by‐breath inspiratory effort and is recognised clinically as a useful index of diaphragm function and respiratory mechanics (Lozano‐Garcia et al., 2019; Lozano‐García et al., 2021), which can guide mechanical ventilatory support to minimise diaphragm dysfunction from ventilator over‐ and under‐assist (Jansen et al., 2021, 2018; Tagliabue et al., 2019). NME is used to observe the level of neuromechanical uncoupling and related breathlessness observed in obstructive lung disease, such as COPD (O'Donnell et al., 2006) and neuromuscular disease (Lanini et al., 2001; Spinelli et al., 1992).

Inspiratory pressure and respiratory EMG values were determined across all states and represented as pressure generated per µV s of integrated EMG activity (cmH_2_O/µV s) to obtain values for NME as shown in the equation below.

$$\mathrm{NME}\;=\frac{\mathrm{Pressure}}{\mathrm{EMG}}$$

#### 2.5.3 Tension time index

The tension–time index (TTI) reflects the efficiency of the total work undertaken by all respiratory muscles. Elevated TTI is associated with inspiratory muscle fatigue (Bellemare & Grassino, 1982; Dassios & Dimitriou, 2019; Miller & Mayer, 2021). TTI has been used to investigate ventilator weaning, severity of lung disease in cystic fibrosis (Hahn et al., 2008) and various neuromuscular diseases including DMD (García‐Río et al., 2003; Hahn et al., 2009; Miller & Mayer, 2021).

Inspiratory pressure, inspiratory time and total duty cycle were calculated across all behaviours and represented as the TTI as shown in the equation below.

$$\mathrm{TTI}=\frac{P}{\mathrm{MIP}}\times \frac{T_{i}}{T_{\mathrm{tot}}}$$

### 2.6 Inspiratory drive rate and EMG rise time

Airway occlusion pressure (*P*_0.1_) is a measure used in mechanically ventilated patients to determine their respiratory drive (Telias et al., 2018). *P*_0.1_ is the pressure developed in the occluded airway 100 ms after the onset of inspiration (Whitelaw et al., 1975). Although it has been used to measure patients’ respiratory drive, *P*_0.1_ is also incorporated in the measure of TTI and has been assessed in patients with cystic fibrosis (Hahn et al., 2008) and DMD (García‐Río et al., 2003; Hahn et al., 2009). The slope of the pressure generated reflects the rate of inspiratory muscle activation and the rate of increase in the electrical activity of the obligatory muscles reflects the rate of activation of the individual muscles also referred to as rise time, which has also been used as an indicator of drive (Telias et al., 2020).

The slope of the inspiratory pressure generation and inspiratory integrated EMG for diaphragm, EIC and PS muscles was generated from onset to peak of the inspiratory time and represented as the inspiratory drive rate and EMG rise times.

#### 2.6.1 Composite pressure–time relationship during sustained airway occlusion

Maximum inspiratory pressure (MIP) is in principle a valuable parameter that can monitor respiratory disease progression, evaluate treatment efficacy and predict patient outcome. In DMD patients, MIP declines at a rate of ∼2.1 cmH_2_O/year generally after the age of 10 years, which generally coincides with loss of ambulation and the beginning of wheelchair dependence (Fauroux et al., 2015; Khirani et al., 2014; van Oosten et al., 2024; Whitelaw et al., 1975). Volitional and non‐invasive assessment of MIP clinically assesses mouth pressure during a maximal inspiratory effort against an occluded mouthpiece (ATS/ERS Statement on Respiratory Muscle Testing, 2002).

Successive inspiratory efforts were measured for the full duration of the single sustained tracheal occlusion event generating maximum inspiratory effort until task failure defined as the inability to sustain peak inspiratory pressure. Each inspiratory effort from the onset of airway occlusion to task failure was measured and represented as a single point on a pressure–time plot. The area under the curve (AUC) of the cumulative pressure–time relationship was evaluated.

#### 2.6.2 EMG signal processing and frequency analysis

EMG signals were sampled at 20 kHz using a custom acquisition system and stored as text files containing time and amplitude data. The analysis pipeline was implemented in MATLAB (MathWorks, Natick, MA, USA) to systematically process all files from a target directory. Each recording contained data from up to three respiratory muscles: diaphragm, external intercostal and parasternal intercostal.

Raw EMG signals underwent a digital filtering approach applied to isolate EMG frequency components: A 50 Hz notch filter (*Q*‐factor = 35) removed power line interference, and a fourth‐order Butterworth bandpass filter (30–1000 Hz) isolated the physiologically relevant EMG frequency range. Filter stability was automatically verified, with second‐order section (SOS) implementations used as fallbacks when traditional implementations proved unstable.

#### 2.6.3 Frequency spectrum analysis

Frequency content analysis was performed using Welch's method for power spectral density estimation. Mean frequency (power‐weighted average frequency across the spectrum), median frequency (frequency dividing the power spectrum into equal halves), maximum frequency, area under the curve (AUC) and total power across the frequency spectrum (30–1000 Hz) were calculated for each muscle at each age group with their corresponding power values. Hamming windows of 2048 samples were used (or maximum available length) with 50% overlap to generate smooth spectral estimates. Trapezoidal numerical integration was employed to calculate the area under the power spectral density curve. The lower frequency limit of 30 Hz was applied to the spectral analysis to effectively exclude ECG artifacts and contamination commonly present at lower frequencies.

### 2.7 Shannon entropy analysis

Shannon entropy (*H*) was calculated to quantify the amplitude distribution complexity of respiratory muscle EMG signals. Signals were filtered for artifacts and baseline drift. Only finite values with a minimum length of 50 samples were analysed. Amplitude values were discretised to three decimal places (precision factor 10^3^). Shannon entropy was computed using the formula:


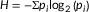

where $p_{i}$ is the probability of occurrence of each unique amplitude value.

### 2.8 Approximate entropy analysis

Approximate entropy (ApEn) was calculated with embedding dimension m=2 and tolerance r=0.25×SD, with SD as the standard deviation of the signal. Signals with at least 200 data points, after preprocessing for stationarity and finite values, were analysed using the standard ApEn algorithm.

### 2.9 Sample entropy analysis

Sample entropy (SampEn) was calculated using m=2 and r=0.25×SD, optimised for mouse respiratory signals. SampEn excludes self‐matches and was computed as:

$$\mathrm{SampEn}\;\left(m,r,N\right)=-\ln\left(\frac{A}{B}\right)$$

where A and B represent counts of matched template vector pairs of lengths m+1 and m, respectively. Signals required a minimum of 200 data points and were preprocessed to remove artifacts.

All computations were implemented in MATLAB with automated batch processing.

#### 2.9.1 Statistical analysis

Values are expressed as box and whisker plots (median, inter‐quartile range (IQR) and individual data scatter plot) in graphs. Data were statistically compared using Prism 10.4.1 (GraphPad Software, Boston, MA, USA). Data for inspiratory (oesophageal) pressure and EMG activities during baseline and tracheal occlusion, and EMG measures during baseline and stable peak levels in vagotomised mice and subsequent chemo‐activation were statistically compared by two‐way mixed ANOVA (time × genotype) with Šidák's multiple comparisons *post hoc* test. Exact *P*‐values are reported for all comparisons. *P* < 0.05 was considered statistically significant.

## 3 RESULTS

### 3.1 Neural respiratory drive

**FIGURE 1:**
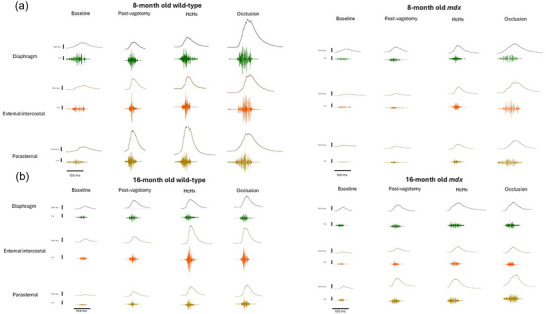
Original traces of individual raw EMG bursts with integrated signals in diaphragm, external intercostal, and parasternal in 8‐month‐old (a) and 16‐month‐old (b) wild‐type and *mdx* mice during baseline, following bilateral vagotomy and during subsequent exposure to hypercapnic hypoxia (HcHx), and sustained tracheal occlusion. Note that electrical activation of the respiratory muscles increases successively across behaviours characterised by increased neural drive to maximum (occlusion). The pattern is similar in wild‐type and *mdx* mice, but the overall level of electrical activation of the respiratory muscles is lower in *mdx* mice compared to wild‐type.

**FIGURE 2:**
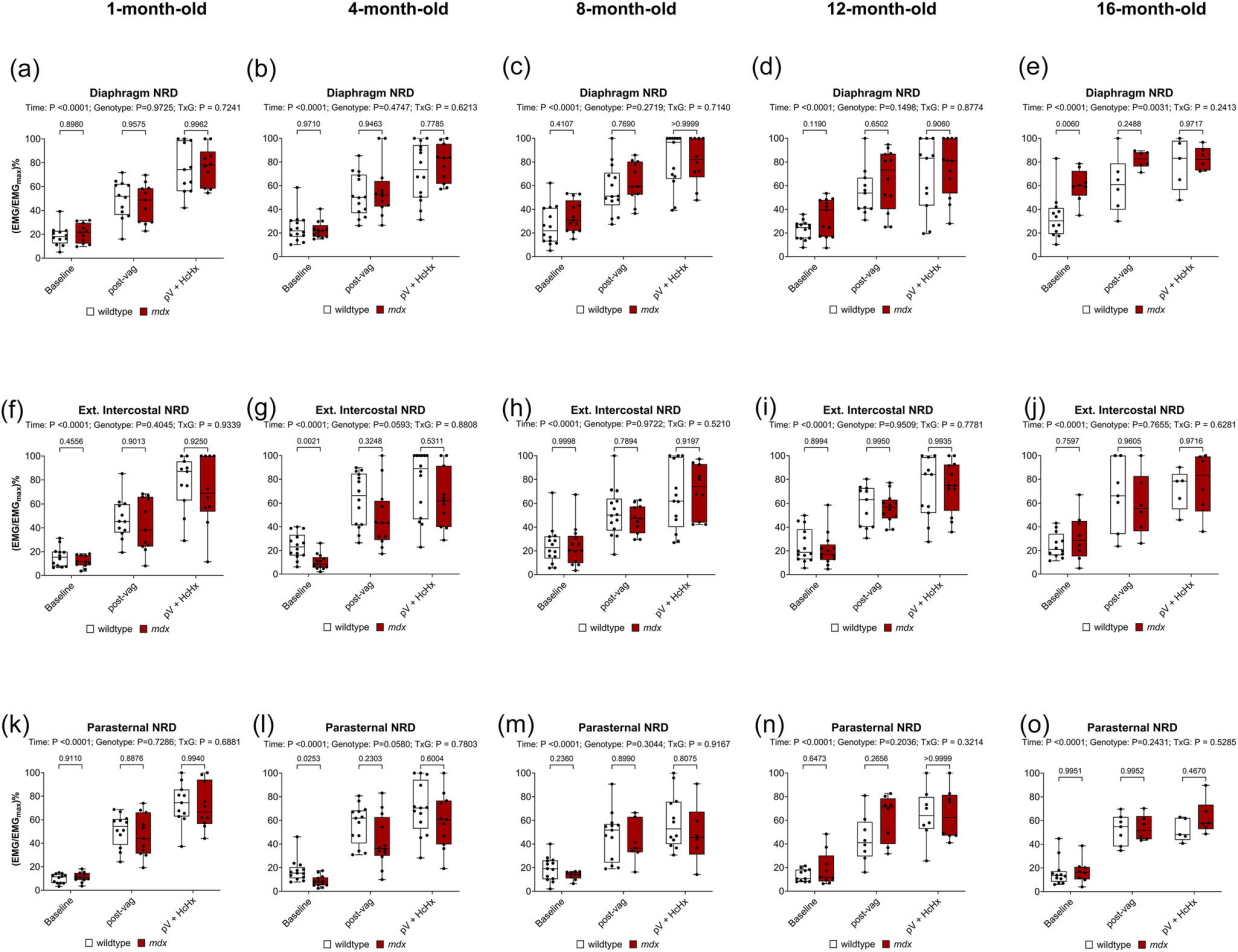
Respiratory muscle neural respiratory drive (NRD) during baseline, following bilateral vagotomy and during subsequent exposure to hypercapnic hypoxia (HcHx) in anaesthetised wild‐type and *mdx* mice. Group data for diaphragm (a–e), external intercostal (f–j) and parasternal intercostal (k–o) NRD during baseline, post‐bilateral vagotomy and during subsequent exposure to HcHx in wild‐type and *mdx* mice at 1, 4, 8, 12 and 16 months of age. Values are expressed as box (median, IQR and individual data points) and whisker (min to max) plots. Data were statistically compared by two‐way mixed ANOVA with Šidák's multiple comparisons *post hoc* test. Absolute *P*‐values for all comparisons are reported.

Original respiratory EMG recordings are shown across all behaviours in Figure 1. The overall level of EMG activity was significantly lower in *mdx* mice in all three respiratory muscles across most behaviours. Figure 2 shows summary EMG data represented as neural respiratory drive (NRD) in anaesthetised wild‐type and *mdx* mice during baseline, following bilateral vagotomy and during subsequent exposure to hypercapnic hypoxia (HcHx). NRD for diaphragm (Figure 2a–e), EIC (Figure 2f–j) and PS (Figure 2k–o) are shown.

For NRD, there was a main effect of time (*P <* 0.0001) across all age groups for all three muscles revealing the expected progressive recruitment of the respiratory muscles with increased respiratory demand.

NRD was significantly higher in *mdx* mice compared to wild‐type mice during baseline conditions at 16 months of age. However, for the most part, NRD of the respiratory muscles was essentially equivalent in wild‐type and *mdx* mice. In summary, there are no major differences between wild‐type and *mdx* mice in NRD in the major muscles of breathing from early to advanced disease.

#### 3.1.1 Frequency spectrum analysis

**FIGURE 3:**
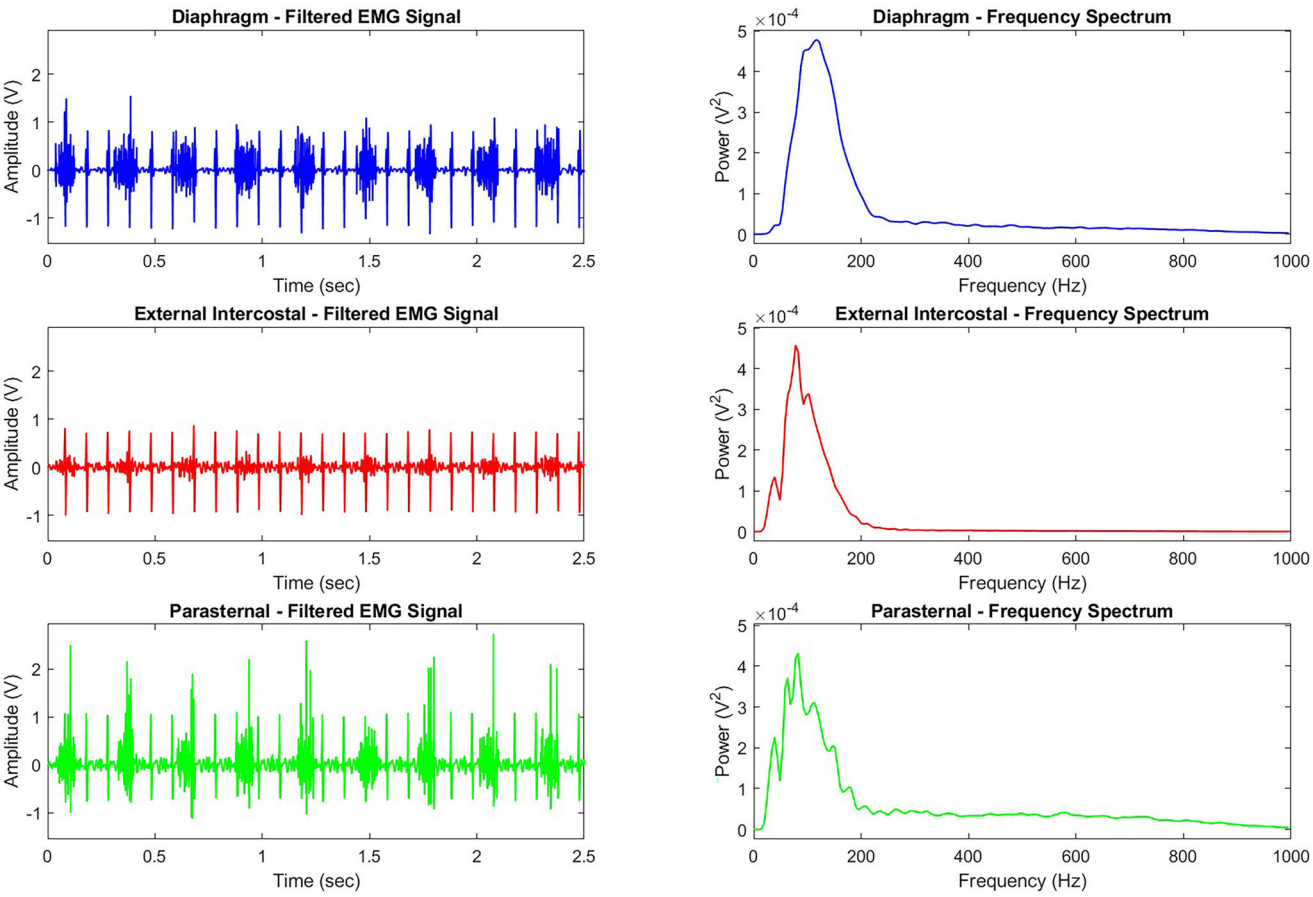
Original traces of filtered EMG signals with frequency spectrum in diaphragm, external intercostal and parasternal in a 1‐month‐old wild‐type mouse during baseline breathing. Mean frequency, median frequency, maximum frequency and area under curve (AUC), were calculated for each muscle at each age group with their corresponding power values.

Filtered respiratory EMG recordings and respective frequency spectrum for diaphragm, EIC and PS in a 1‐month‐old wild‐type mouse are shown in Figure 3. Mean frequency, median frequency, maximum frequency and area under the curve (AUC) were calculated for each muscle at each age group with corresponding power values and data are shown in Table 1.

**TABLE 1:** Frequency spectrum parameters for inspiratory muscle EMG activities in urethane‐anaesthetised wild‐type and *mdx* mice.

Two‐way ANOVA	Diaphragm			External intercostal			Parasternal		
	Time	Genotype	Time × genotype	Time	Genotype	Time × genotype	Time	Genotype	Time × genotype
1 month old									
Mean frequency (Hz)	<0.0001	0.0309	0.4327	<0.0001	0.0591	0.2703	<0.0001	0.0023	0.0268
Mean power (mV^2^)	0.0075	0.953	0.1442	0.0257	0.0043	0.0519	0.0028	0.0696	0.0038
Median frequency (Hz)	<0.0001	0.0462	0.1057	<0.0001	0.0756	0.0999	<0.0001	0.0012	0.0009
Median power (mV^2^)	0.0071	0.2552	0.427	0.0175	0.0867	0.0579	0.0017	0.0464	0.0393
Max frequency (Hz)	0.0492	0.0518	0.0059	0.0002	0.5415	0.5374	0.0096	0.5282	0.4453
Max power (mV^2^)	0.2928	0.2838	0.5714	0.1174	0.0019	0.1125	0.0008	0.1776	0.2011
Total power (mV^2^)	0.014	0.4434	0.485	0.0195	0.0319	0.0164	0.0976	0.8778	0.0105
4 months old									
Mean frequency (Hz)	<0.0001	0.0071	0.4833	<0.0001	0.9991	0.551	<0.0001	0.4713	0.9988
Mean power (mV^2^)	0.1457	0.4796	0.0008	0.0029	0.9888	0.2129	0.0008	0.7241	0.6136
Median frequency (Hz)	<0.0001	0.0305	0.2197	<0.0001	0.3659	0.4755	<0.0001	0.5712	0.9897
median power (mV^2^)	0.3033	0.543	0.1917	<0.0001	0.2327	0.3475	<0.0001	0.0892	0.7828
Max frequency (Hz)	0.0038	0.0019	<0.0001	0.0226	0.9885	0.2153	0.0021	0.1587	0.0006
Max power (mV^2^)	0.0784	0.661	0.0891	0.001	0.1332	0.743	0.3333	0.2026	0.8904
Total power (mV^2^)	0.0061	0.0456	0.0038	0.6955	0.0581	0.9858	0.0002	0.1653	0.6102
8 months old									
Mean frequency (Hz)	<0.0001	0.003	0.0004	<0.0001	0.0062	0.9449	<0.0001	0.0004	0.0877
Mean power (mV^2^)	0.0443	0.0619	0.0014	0.0147	0.495	0.0024	0.287	0.0199	0.0882
Median frequency (Hz)	0.0002	0.0018	<0.0001	<0.0001	0.0068	0.2587	<0.0001	0.0006	0.2972
Median power (mV^2^)	0.0076	0.7086	<0.0001	0.0219	0.0482	<0.0001	0.8768	0.0248	0.0143
Max frequency (Hz)	0.0423	0.0271	0.0005	0.0003	0.0025	0.0001	0.0079	0.0006	0.0194
Max power (mV^2^)	0.037	0.5603	<0.0001	0.1642	0.5626	0.0216	0.1559	0.0164	0.0147
Total power (mV^2^)	0.0022	0.0197	0.0125	0.0006	0.1984	0.0116	0.0022	0.0029	0.0009
12 months old									
Mean frequency (Hz)	<0.0001	0.0003	0.2089	<0.0001	0.8205	0.7289	<0.0001	0.1269	0.3223
Mean power (mV^2^)	0.0038	0.3941	0.0068	0.1308	0.4046	0.0576	0.0832	0.0071	0.0847
Median frequency (Hz)	<0.0001	0.0003	0.059	<0.0001	0.8058	0.8593	<0.0001	0.2218	0.313
Median power (mV^2^)	0.8031	0.1983	0.6739	<0.0001	0.2225	0.6208	0.1494	0.0084	0.2275
Max frequency (Hz)	0.0036	0.0018	<0.0001	0.0007	0.8475	0.9789	0.1231	0.1604	0.44
Max power (mV^2^)	0.748	0.2819	0.3448	0.0251	0.0788	0.5467	0.5119	0.0036	0.2559
Total power (mV^2^)	0.0348	0.06	0.0032	0.0682	0.0153	0.4615	0.3687	0.0015	0.8173
16 months old									
Mean frequency (Hz)	0.0007	0.1059	0.0448	<0.0001	0.2452	0.8933	<0.0001	0.7591	0.6226
Mean power (mV^2^)	0.1159	0.0634	0.0733	0.8186	0.0089	0.1513	0.2986	0.0131	0.0943
Median frequency (Hz)	0.0044	0.0795	0.1819	<0.0001	0.3581	0.6055	0.0002	0.6819	0.9903
Median power (mV^2^)	0.2052	0.0528	0.5935	0.236	0.0278	0.3144	0.0072	0.0553	0.1192
Max frequency (Hz)	0.0565	0.4561	0.1366	0.0573	0.0713	0.0468	0.0053	0.6521	0.0622
Max power (mV^2^)	0.1165	0.0198	0.2568	0.7353	0.0209	0.9931	0.0112	0.2529	0.2326
Total power (mV^2^)	0.1384	0.0094	0.1749	0.0987	0.0172	0.0494	0.2764	0.124	0.9317

	Diaphragm			External intercostal			Parasternal		
	Mean ± SD		Adjusted *P*‐value	Mean ± SD		Adjusted *P*‐value	Mean ± SD		Adjusted *P*‐value
	Wild‐type	*mdx*		Wild‐type	*mdx*		Wild‐type	*mdx*	
1 month old									
Baseline									
*n*	12	11		12	11		12	11	
Mean frequency (Hz)	178 ± 35	159 ± 40	0.6865	162 ± 27	135 ± 15	0.0468	164 ± 54	130 ± 23	0.2247
Mean power (mV^2^)	0.25 ± 0.31	0.20 ± 0.19	0.9842	0.12 ± 0.11	0.12 ± 0.08	0.9999	0.43 ± 0.38	1.53 ± 1.62	0.1856
Median frequency (Hz)	123 ± 22	123 ± 17	>0.9999	108 ± 13	112 ± 18	0.9505	113 ± 24	107 ± 12	0.8751
Median power (mV^2^)	0.49 ± 0.45	0.29 ± 0.18	0.5965	0.49 ± 0.42	0.24 ± 0.20	0.3076	0.82 ± 0.57	1.90 ± 1.91	0.3353
Max frequency (Hz)	98 ± 20	95 ± 14	0.9898	83 ± 15	95 ± 12	0.1691	87 ± 20	96 ± 7	0.4996
Max power (mV^2^)	0.57 ± 0.50	0.41 ± 0.30	0.8618	0.67 ± 0.57	0.28 ± 0.21	0.1735	1.12 ± 0.65	2.29 ± 2.29	0.4249
Total power (mV^2^)	52.56 ± 33.02	53.93 ± 39.65	>0.9999	65.54 ± 58.34	30.92 ± 22.13	0.2761	98.71 ± 42.31	199.92 ± 206.51	0.4520
Vagotomy									
*n*	12	11		12	11		12	11	
Mean frequency (Hz)	214 ± 84	155 ± 29	0.1480	267 ± 100	197 ± 57	0.1884	243 ± 84	141 ± 18	0.0057
Mean power (mV^2^)	0.16 ± 0.17	0.24 ± 0.20	0.8098	0.08 ± 0.06	0.03 ± 0.02	0.1487	0.29 ± 0.26	0.93 ± 1.01	0.2394
Median frequency (Hz)	132 ± 35	120 ± 10	0.7138	218 ± 111	130 ± 33	0.0843	166 ± 68	111 ± 10	0.0623
Median power (mV^2^)	0.39 ± 0.29	0.28 ± 0.20	0.8122	0.12 ± 0.08	0.19 ± 0.27	0.8985	0.50 ± 0.34	1.71 ± 1.68	0.1467
Max frequency (Hz)	104 ± 11	98 ± 11	0.6600	89 ± 13	95 ± 16	0.8177	95 ± 8	96 ± 7	0.9854
Max power (mV^2^)	0.50 ± 0.32	0.33 ± 0.21	0.5151	0.27 ± 0.18	0.17 ± 0.20	0.7494	1.29 ± 0.73	2.03 ± 1.86	0.6657
Total power (mV^2^)	71.05 ± 45.01	60.72 ± 48.77	0.9771	50.99 ± 34.38	33.57 ± 41.64	0.7666	168.73 ± 85.24	187.58 ± 158.61	0.9947
Asphyxia									
*n*	10	10		10	10		10	10	
Mean frequency (Hz)	260 ± 100	195 ± 51	0.3058	334 ± 95	247 ± 72	0.1362	272 ± 78	193 ± 69	0.1055
Mean power (mV^2^)	0.09 ± 0.06	0.22 ± 0.19	0.2467	0.12 ± 0.08	0.08 ± 0.07	0.7413	0.28 ± 0.23	0.27 ± 0.21	0.9999
Median frequency (Hz)	199 ± 125	135 ± 25	0.4741	289 ± 114	188 ± 80	0.1385	193 ± 81	112 ± 17	0.0447
Median power (mV^2^)	0.35 ± 0.28	0.24 ± 0.15	0.8179	0.14 ± 0.08	0.14 ± 0.16	>0.9999	0.49 ± 0.31	1.54 ± 1.64	0.2749
Max frequency (Hz)	101 ± 22	100 ± 14	>0.9999	119 ± 48	103 ± 30	0.8679	90 ± 16	91 ± 9	>0.9999
Max power (mV^2^)	0.58 ± 0.32	0.36 ± 0.20	0.4019	0.27 ± 0.14	0.24 ± 0.24	0.9979	1.43 ± 0.80	2.03 ± 1.94	0.8576
Total power (mV^2^)	99.80 ± 56.34	91.23 ± 80.08	0.9980	77.64 ± 54.47	82.83 ± 88.13	0.9998	237.29 ± 137.48	202.05 ± 57.96	0.9747
Occlusion									
*n*	12	11		12	11		12	11	
Mean frequency (Hz)	339 ± 102	268 ± 85	0.2974	379 ± 105	335 ± 75	0.7013	413 ± 54	308 ± 80	0.0071
Mean power (mV^2^)	0.13 ± 0.09	0.06 ± 0.03	0.0860	0.21 ± 0.12	0.07 ± 0.05	0.0111	0.17 ± 0.09	0.17 ± 0.16	>0.9999
Median frequency (Hz)	297 ± 131	203 ± 106	0.2577	348 ± 136	297 ± 96	0.7723	387 ± 78	252 ± 104	0.0097
Median power (mV^2^)	0.20 ± 0.12	0.19 ± 0.15	>0.9999	0.29 ± 0.22	0.08 ± 0.06	0.0281	0.28 ± 0.19	0.26 ± 0.23	0.9997
Max frequency (Hz)	223 ± 182	95 ± 54	0.1394	250 ± 154	205 ± 135	0.9129	188 ± 144	143 ± 68	0.8173
Max power (mV^2^)	0.44 ± 0.29	0.42 ± 0.30	0.9996	0.62 ± 0.55	0.14 ± 0.07	0.0451	0.56 ± 0.44	0.76 ± 0.69	0.9036
Total power (mV^2^)	101.52 ± 55.53	73.11 ± 65.35	0.7391	143.10 ± 96.28	41.93 ± 30.44	0.0165	189.95 ± 144.94	143.44 ± 127.87	0.8890
4 months old									
Baseline									
*n*	14	13		14	13		12	12	
Mean frequency (Hz)	278 ± 88	147 ± 29	0.0003	167 ± 28	162 ± 36	0.9900	173 ± 20	154 ± 37	0.5098
Mean power (mV^2^)	0.08 ± 0.06	0.18 ± 0.20	0.4558	0.23 ± 0.17	0.35 ± 0.40	0.8380	0.32 ± 0.19	0.38 ± 0.43	0.9850
Median frequency (Hz)	225 ± 92	114 ± 14	0.0022	115 ± 13	122 ± 12	0.4477	125 ± 11	112 ± 12	0.0510
Median power (mV^2^)	0.10 ± 0.09	0.17 ± 0.15	0.5947	0.58 ± 0.35	0.44 ± 0.42	0.8479	0.70 ± 0.28	0.56 ± 0.49	0.8610
Max frequency (Hz)	86 ± 18	83 ± 22	0.9944	85 ± 22	92 ± 19	0.8905	104 ± 14	97 ± 16	0.7551
Max power (mV^2^)	0.26 ± 0.17	0.22 ± 0.20	0.9833	0.76 ± 0.42	0.51 ± 0.45	0.5061	0.86 ± 0.35	0.63 ± 0.54	0.6328
Total power (mV^2^)	47.77 ± 25.22	41.24 ± 41.89	0.9862	80.30 ± 45.39	56.80 ± 53.80	0.6769	95.35 ± 48.77	61.28 ± 55.42	0.4122
Vagotomy									
*n*	14	12		14	12		12	12	
Mean frequency (Hz)	315 ± 106	240 ± 94	0.2402	260 ± 85	237 ± 106	0.9612	265 ± 67	257 ± 87	0.9986
Mean power (mV^2^)	0.04 ± 0.03	0.13 ± 0.14	0.2259	0.09 ± 0.06	0.04 ± 0.03	0.0761	0.13 ± 0.12	0.08 ± 0.06	0.5878
Median frequency (Hz)	250 ± 145	182 ± 89	0.4900	160 ± 54	154 ± 63	0.9982	154 ± 30	136 ± 17	0.3306
Median power (mV^2^)	0.19 ± 0.17	0.19 ± 0.19	>0.9999	0.29 ± 0.19	0.31 ± 0.33	0.9994	0.41 ± 0.34	0.15 ± 0.11	0.1067
Max frequency (Hz)	94 ± 21	82 ± 26	0.6333	92 ± 28	103 ± 23	0.7579	107 ± 12	91 ± 19	0.1103
Max power (mV^2^)	0.30 ± 0.23	0.30 ± 0.29	>0.9999	0.58 ± 0.25	0.46 ± 0.42	0.9076	0.69 ± 0.43	0.49 ± 0.44	0.7385
Total power (mV^2^)	57.51 ± 37.22	52.20 ± 58.90	0.9983	86.06 ± 30.45	54.11 ± 48.00	0.2589	108.98 ± 82.60	78.21 ± 67.88	0.7986
Asphyxia									
*n*	14	11		14	11		12	11	
Mean frequency (Hz)	364 ± 85	264 ± 120	0.1224	292 ± 83	310 ± 128	0.9916	302 ± 70	292 ± 69	0.9938
Mean power (mV^2^)	0.07 ± 0.05	0.11 ± 0.10	0.7922	0.12 ± 0.09	0.05 ± 0.03	0.0591	0.14 ± 0.11	0.06 ± 0.05	0.2145
Median frequency (Hz)	302 ± 142	213 ± 135	0.4164	212 ± 98	270 ± 144	0.7108	214 ± 82	217 ± 95	>0.9999
Median power (mV^2^)	0.15 ± 0.12	0.22 ± 0.24	0.8643	0.27 ± 0.19	0.08 ± 0.08	0.0173	0.34 ± 0.28	0.16 ± 0.14	0.2197
Max frequency (Hz)	93 ± 18	81 ± 21	0.5618	94 ± 32	91 ± 18	0.9966	116 ± 22	87 ± 17	0.0070
Max power (mV^2^)	0.36 ± 0.25	0.34 ± 0.37	0.9997	0.52 ± 0.18	0.38 ± 0.45	0.8599	0.77 ± 0.53	0.42 ± 0.40	0.3014
Total power (mV^2^)	91.24 ± 59.55	53.13 ± 39.77	0.2635	88.60 ± 32.35	65.13 ± 60.85	0.7448	120.22 ± 88.10	59.83 ± 45.89	0.2329
Occlusion									
*n*	14	13		14	12		13	13	
Mean frequency (Hz)	424 ± 117	290 ± 140	0.0505	362 ± 91	390 ± 110	0.9324	387 ± 87	372 ± 109	0.9919
Mean power (mV^2^)	0.22 ± 0.22	0.08 ± 0.06	0.1787	0.11 ± 0.09	0.08 ± 0.05	0.7191	0.25 ± 0.24	0.22 ± 0.20	0.9979
Median frequency (Hz)	404 ± 160	245 ± 172	0.0806	313 ± 126	363 ± 143	0.8278	349 ± 116	338 ± 150	0.9992
Median power (mV^2^)	0.26 ± 0.25	0.21 ± 0.20	0.9632	0.20 ± 0.15	0.08 ± 0.05	0.0719	0.27 ± 0.23	0.23 ± 0.24	0.9953
Max frequency (Hz)	341 ± 263	94 ± 20	0.0152	119 ± 37	104 ± 16	0.6906	140 ± 33	301 ± 224	0.0953
Max power (mV^2^)	0.57 ± 0.51	0.33 ± 0.29	0.4651	0.38 ± 0.29	0.24 ± 0.15	0.4414	0.79 ± 0.64	0.65 ± 0.75	0.9793
Total power (mV^2^)	165.65 ± 137.10	55.80 ± 32.90	0.0437	89.11 ± 52.63	69.98 ± 50.78	0.8372	234.78 ± 199.80	155.18 ± 140.93	0.6891
8 months old									
Baseline									
*n*	15	14		15	14		14	10	
Mean frequency (Hz)	243 ± 85	244 ± 83	>0.9999	241 ± 94	159 ± 41	0.0240	166 ± 34	142 ± 21	0.1668
Mean power (mV^2^)	0.07 ± 0.08	0.04 ± 0.03	0.5451	0.06 ± 0.06	0.11 ± 0.08	0.2584	0.24 ± 0.15	0.14 ± 0.18	0.5839
Median frequency (Hz)	155 ± 60	191 ± 75	0.5384	141 ± 64	111 ± 17	0.3676	125 ± 21	101 ± 22	0.0586
Median power (mV^2^)	0.10 ± 0.09	0.05 ± 0.04	0.4045	0.06 ± 0.04	0.23 ± 0.09	<0.0001	0.45 ± 0.22	0.28 ± 0.26	0.4057
Max frequency (Hz)	83 ± 15	103 ± 34	0.2449	72 ± 13	86 ± 12	0.0278	99 ± 18	85 ± 29	0.5335
Max power (mV^2^)	0.29 ± 0.30	0.09 ± 0.04	0.1003	0.35 ± 0.29	0.35 ± 0.13	>0.9999	0.58 ± 0.27	0.28 ± 0.16	0.0179
Total power (mV^2^)	25.70 ± 22.60	16.16 ± 6.16	0.5553	44.20 ± 33.61	29.88 ± 13.39	0.4678	68.97 ± 42.74	26.45 ± 13.50	0.0170
Vagotomy									
*n*	16	14		16	14		15	11	
Mean frequency (Hz)	323 ± 106	239 ± 100	0.1291	330 ± 141	241 ± 84	0.1578	232 ± 57	163 ± 53	0.0154
Mean power (mV^2^)	0.05 ± 0.04	0.07 ± 0.07	0.8303	0.03 ± 0.02	0.05 ± 0.05	0.6689	0.14 ± 0.10	0.11 ± 0.10	0.8883
Median frequency (Hz)	278 ± 141	193 ± 103	0.2420	279 ± 176	175 ± 85	0.1749	164 ± 55	107 ± 33	0.0139
Median power (mV^2^)	0.09 ± 0.08	0.19 ± 0.18	0.2994	0.04 ± 0.03	0.19 ± 0.19	0.0832	0.51 ± 0.37	0.28 ± 0.24	0.2534
Max frequency (Hz)	82 ± 17	108 ± 32	0.0889	77 ± 18	82 ± 16	0.9128	104 ± 19	74 ± 14	0.0004
Max power (mV^2^)	0.12 ± 0.08	0.23 ± 0.19	0.2311	0.27 ± 0.26	0.46 ± 0.38	0.4483	0.73 ± 0.46	0.39 ± 0.25	0.1196
Total power (mV^2^)	31.88 ± 20.17	32.01 ± 16.57	>0.9999	42.72 ± 27.67	55.11 ± 37.76	0.7880	129.22 ± 103.92	35.18 ± 20.82	0.0148
Asphyxia									
*n*	14	12		14	12		13	9	
Mean frequency (Hz)	386 ± 102	211 ± 87	0.0003	412 ± 130	307 ± 62	0.0553	261 ± 55	153 ± 48	0.0004
Mean power (mV^2^)	0.11 ± 0.10	0.07 ± 0.05	0.7033	0.07 ± 0.05	0.04 ± 0.03	0.5472	0.18 ± 0.18	0.08 ± 0.06	0.2518
Median frequency (Hz)	348 ± 159	104 ± 23	0.0003	392 ± 171	243 ± 91	0.0414	183 ± 62	92 ± 25	0.0008
Median power (mV^2^)	0.12 ± 0.14	0.31 ± 0.27	0.1782	0.06 ± 0.04	0.06 ± 0.04	0.9986	0.41 ± 0.32	0.37 ± 0.34	0.9971
Max frequency (Hz)	233 ± 207	82 ± 24	0.0683	245 ± 236	85 ± 28	0.0987	109 ± 19	65 ± 8	<0.0001
Max power (mV^2^)	0.13 ± 0.09	0.43 ± 0.29	0.0183	0.20 ± 0.17	0.31 ± 0.28	0.6961	0.64 ± 0.41	0.56 ± 0.42	0.9867
Total power (mV^2^)	92.82 ± 96.37	44.73 ± 13.39	0.3044	54.07 ± 40.52	45.65 ± 32.24	0.9653	129.77 ± 96.75	59.84 ± 53.68	0.1607
Occlusion									
*n*	16	14		15	13		15	11	
Mean frequency (Hz)	386 ± 103	306 ± 89	0.1213	450 ± 90	371 ± 61	0.0408	371 ± 105	297 ± 74	0.1677
Mean power (mV^2^)	0.15 ± 0.15	0.04 ± 0.02	0.0417	0.17 ± 0.15	0.07 ± 0.05	0.1210	0.34 ± 0.32	0.09 ± 0.09	0.0615
Median frequency (Hz)	363 ± 127	247 ± 114	0.0529	435 ± 112	331 ± 101	0.0633	334 ± 138	241 ± 96	0.2025
Median power (mV^2^)	0.25 ± 0.23	0.06 ± 0.04	0.0256	0.18 ± 0.14	0.08 ± 0.08	0.1241	0.72 ± 0.69	0.07 ± 0.04	0.0117
Max frequency (Hz)	166 ± 94	108 ± 31	0.1945	366 ± 247	106 ± 30	0.0045	258 ± 199	107 ± 46	0.0481
Max power (mV^2^)	0.45 ± 0.37	0.16 ± 0.11	0.0334	0.38 ± 0.36	0.16 ± 0.06	0.1491	1.14 ± 0.97	0.31 ± 0.26	0.0240
Total power (mV^2^)	121.49 ± 118.43	34.79 ± 21.41	0.0430	132.87 ± 113.76	66.13 ± 44.36	0.1890	268.88 ± 202.80	52.13 ± 34.27	0.0041
12 months old									
Baseline									
*n*	14	13		14	13		12	9	
Mean frequency (Hz)	319 ± 119	220 ± 69	0.0564	200 ± 49	188 ± 51	0.9558	179 ± 52	239 ± 80	0.2543
Mean power (mV^2^)	0.06 ± 0.06	0.11 ± 0.11	0.5617	0.05 ± 0.04	0.10 ± 0.11	0.5138	0.28 ± 0.28	0.06 ± 0.06	0.1271
Median frequency (Hz)	273 ± 159	171 ± 68	0.1540	120 ± 19	115 ± 15	0.9016	126 ± 21	187 ± 63	0.0782
Median power (mV^2^)	0.17 ± 0.19	0.12 ± 0.06	0.8613	0.24 ± 0.20	0.22 ± 0.17	0.9963	0.76 ± 0.76	0.09 ± 0.09	0.0604
Max frequency (Hz)	90 ± 13	93 ± 27	0.9953	81 ± 13	88 ± 10	0.4773	98 ± 6	112 ± 45	0.8309
Max power (mV^2^)	0.29 ± 0.28	0.25 ± 0.18	0.9898	0.37 ± 0.24	0.27 ± 0.14	0.5547	0.94 ± 0.82	0.09 ± 0.08	0.0256
Total power (mV^2^)	45.89 ± 33.84	51.27 ± 39.81	0.9929	42.98 ± 30.52	28.27 ± 16.79	0.4342	96.17 ± 74.15	18.54 ± 19.10	0.0244
Vagotomy									
*n*	12	12		12	12		10	8	
Mean frequency (Hz)	331 ± 86	229 ± 56	0.0108	264 ± 85	265 ± 99	>0.9999	243 ± 109	330 ± 88	0.2806
Mean power (mV^2^)	0.05 ± 0.03	0.05 ± 0.02	>0.9999	0.04 ± 0.04	0.02 ± 0.01	0.6390	0.11 ± 0.08	0.04 ± 0.03	0.1695
Median frequency (Hz)	276 ± 134	151 ± 21	0.0323	155 ± 63	146 ± 54	0.9949	182 ± 98	272 ± 106	0.2960
Median power (mV^2^)	0.14 ± 0.12	0.14 ± 0.10	>0.9999	0.17 ± 0.18	0.11 ± 0.09	0.8182	0.71 ± 0.85	0.06 ± 0.06	0.1894
Max frequency (Hz)	95 ± 16	96 ± 29	>0.9999	81 ± 18	85 ± 11	0.9523	97 ± 10	159 ± 129	0.6172
Max power (mV^2^)	0.29 ± 0.19	0.23 ± 0.15	0.8955	0.32 ± 0.25	0.21 ± 0.14	0.6791	0.98 ± 1.00	0.11 ± 0.08	0.1176
Total power (mV^2^)	58.22 ± 41.98	51.74 ± 36.40	0.9908	60.04 ± 53.37	26.76 ± 12.65	0.2101	109.19 ± 87.89	22.88 ± 13.48	0.0724
Asphyxia									
*n*	12	12		12	12		10	8	
Mean frequency (Hz)	373 ± 109	214 ± 78	0.0021	317 ± 99	322 ± 109	>0.9999	278 ± 121	311 ± 60	0.9189
Mean power (mV^2^)	0.08 ± 0.06	0.04 ± 0.02	0.3087	0.08 ± 0.08	0.03 ± 0.02	0.3666	0.10 ± 0.07	0.03 ± 0.02	0.0964
Median frequency (Hz)	342 ± 155	136 ± 71	0.0030	251 ± 138	256 ± 146	>0.9999	227 ± 153	240 ± 78	0.9990
Median power (mV^2^)	0.17 ± 0.16	0.10 ± 0.04	0.4582	0.14 ± 0.17	0.05 ± 0.04	0.3763	0.50 ± 0.56	0.05 ± 0.04	0.1755
Max frequency (Hz)	94 ± 18	65 ± 9	0.0020	94 ± 18	80 ± 17	0.2359	91 ± 12	85 ± 32	0.9854
Max power (mV^2^)	0.32 ± 0.22	0.25 ± 0.15	0.8595	0.28 ± 0.21	0.17 ± 0.09	0.3709	0.79 ± 0.71	0.14 ± 0.09	0.0983
Total power (mV^2^)	83.78 ± 64.38	42.86 ± 32.28	0.2466	62.91 ± 44.47	28.47 ± 12.95	0.0905	143.22 ± 137.62	26.29 ± 17.64	0.0970
Occlusion									
*n*	14	13		14	13		12	18	
Mean frequency (Hz)	442 ± 92	300 ± 112	0.0061	384 ± 81	360 ± 69	0.8843	355 ± 84	366 ± 74	0.9972
Mean power (mV^2^)	0.15 ± 0.10	0.08 ± 0.05	0.1622	0.10 ± 0.08	0.06 ± 0.04	0.4780	0.09 ± 0.07	0.07 ± 0.08	0.9615
Median frequency (Hz)	430 ± 123	244 ± 124	0.0024	343 ± 116	313 ± 119	0.9445	306 ± 124	319 ± 99	0.9980
Median power (mV^2^)	0.19 ± 0.14	0.13 ± 0.08	0.5283	0.10 ± 0.07	0.06 ± 0.02	0.1876	0.22 ± 0.29	0.03 ± 0.02	0.2671
Max frequency (Hz)	317 ± 230	95 ± 44	0.0134	251 ± 217	238 ± 189	0.9997	123 ± 55	156 ± 140	0.9562
Max power (mV^2^)	0.39 ± 0.33	0.22 ± 0.10	0.3403	0.22 ± 0.14	0.19 ± 0.07	0.9460	0.57 ± 0.58	0.21 ± 0.25	0.2943
Total power (mV^2^)	114.17 ± 84.60	45.47 ± 25.31	0.0546	68.90 ± 45.64	46.20 ± 22.07	0.4126	146.82 ± 151.29	49.64 ± 45.33	0.2416
16 months old									
Baseline									
*n*	12	10		12	10		12	10	
Mean frequency (Hz)	244 ± 129	215 ± 71	0.9458	232 ± 99	197 ± 57	0.7699	158 ± 34	157 ± 50	>0.9999
Mean power (mV^2^)	0.05 ± 0.04	0.05 ± 0.03	0.9991	0.07 ± 0.07	0.03 ± 0.03	0.4252	0.28 ± 0.27	0.07 ± 0.04	0.1483
Median frequency (Hz)	202 ± 139	153 ± 68	0.7507	149 ± 70	141 ± 45	0.9955	119 ± 16	112 ± 35	0.9565
Median power (mV^2^)	0.29 ± 0.39	0.10 ± 0.06	0.4839	0.20 ± 0.20	0.05 ± 0.04	0.141	0.31 ± 0.29	0.22 ± 0.18	0.9209
Max frequency (Hz)	86 ± 15	103 ± 37	0.5766	93 ± 19	88 ± 8	0.9071	93 ± 27	73 ± 21	0.2349
Max power (mV^2^)	0.38 ± 0.41	0.18 ± 0.11	0.4593	0.30 ± 0.29	0.11 ± 0.09	0.2151	1.00 ± 1.07	0.37 ± 0.26	0.2512
Total power (mV^2^)	44.61 ± 33.41	25.44 ± 16.14	0.3746	36.68 ± 25.21	12.92 ± 9.20	0.0475	97.84 ± 96.58	45.17 ± 37.55	0.3526
Vagotomy									
*n*	7	6		7	6		7	6	
Mean frequency (Hz)	252 ± 102	240 ± 116	0.9994	286 ± 126	271 ± 102	0.9987	162 ± 25	191 ± 47	0.6514
Mean power (mV^2^)	0.09 ± 0.07	0.05 ± 0.03	0.7019	0.08 ± 0.06	0.04 ± 0.05	0.7152	0.28 ± 0.17	0.05 ± 0.03	0.0457
Median frequency (Hz)	202 ± 115	192 ± 128	0.9998	239 ± 149	228 ± 104	0.9998	115 ± 19	112 ± 20	0.9992
Median power (mV^2^)	0.25 ± 0.24	0.13 ± 0.12	0.7076	0.15 ± 0.13	0.03 ± 0.03	0.2439	0.69 ± 0.57	0.21 ± 0.15	0.2466
Max frequency (Hz)	91 ± 31	119 ± 49	0.6988	102 ± 15	85 ± 11	0.1684	86 ± 16	80 ± 21	0.9671
Max power (mV^2^)	0.39 ± 0.30	0.11 ± 0.05	0.1624	0.29 ± 0.23	0.13 ± 0.14	0.5506	0.96 ± 0.90	0.74 ± 0.68	0.9793
Total power (mV^2^)	60.68 ± 37.67	24.02 ± 15.02	0.1691	54.08 ± 39.16	14.73 ± 8.52	0.2089	107.68 ± 98.56	66.86 ± 54.53	0.8425
Asphyxia									
*n*	5	5		5	5		5	5	
Mean frequency (Hz)	283 ± 67	246 ± 87	0.9252	345 ± 139	318 ± 82	0.9938	261 ± 102	210 ± 32	0.8065
Mean power (mV^2^)	0.14 ± 0.10	0.04 ± 0.02	0.2891	0.12 ± 0.09	0.02 ± 0.01	0.2229	0.13 ± 0.12	0.08 ± 0.05	0.9326
Median frequency (Hz)	232 ± 99	178 ± 100	0.8792	295 ± 194	282 ± 114	>0.9999	128 ± 12	117 ± 10	0.5818
Median power (mV^2^)	0.23 ± 0.13	0.04 ± 0.01	0.1454	0.13 ± 0.07	0.04 ± 0.03	0.3071	0.38 ± 0.36	0.26 ± 0.19	0.9574
Max frequency (Hz)	103 ± 23	92 ± 27	0.945	103 ± 20	88 ± 43	0.9498	92 ± 11	71 ± 10	0.0627
Max power (mV^2^)	0.46 ± 0.41	0.10 ± 0.03	0.3817	0.28 ± 0.23	0.10 ± 0.08	0.6139	0.54 ± 0.49	0.67 ± 0.44	0.9891
Total power (mV^2^)	102.91 ± 90.80	29.56 ± 24.79	0.4707	66.95 ± 39.40	17.41 ± 12.71	0.2904	73.40 ± 58.79	73.03 ± 47.44	>0.9999
Occlusion									
*n*	12	9		12	9		12	9	
Mean frequency (Hz)	362 ± 123	255 ± 102	0.1554	420 ± 80	355 ± 111	0.4997	368 ± 108	353 ± 73	0.9926
Mean power (mV^2^)	0.07 ± 0.04	0.04 ± 0.02	0.3236	0.12 ± 0.09	0.02 ± 0.01	0.0094	0.11 ± 0.14	0.09 ± 0.09	0.9957
Median frequency (Hz)	335 ± 153	205 ± 104	0.119	403 ± 114	316 ± 145	0.5001	321 ± 154	302 ± 104	0.9947
Median power (mV^2^)	0.10 ± 0.10	0.05 ± 0.04	0.5601	0.10 ± 0.08	0.05 ± 0.06	0.5163	0.22 ± 0.22	0.10 ± 0.10	0.3578
Max frequency (Hz)	234 ± 198	131 ± 65	0.389	311 ± 267	106 ± 42	0.089	107 ± 47	132 ± 42	0.6472
Max power (mV^2^)	0.16 ± 0.09	0.11 ± 0.09	0.7011	0.29 ± 0.25	0.09 ± 0.08	0.0918	0.53 ± 0.60	0.41 ± 0.44	0.9699
Total power (mV^2^)	40.95 ± 17.98	27.78 ± 23.26	0.5711	109.78 ± 99.20	13.51 ± 5.09	0.0252	124.63 ± 136.37	72.16 ± 75.18	0.7374

For mean frequency, there was a main effect of time (*P <* 0.0001) across all age groups for all three muscles reflecting changes in spectral features associated with recruitment of muscles with increased respiratory demand. There were significant differences between wild‐type and *mdx* mice for diaphragm at 1, 4, 8 and 12 months (*P* = 0.0309, *P* = 0.0071, *P* = 0.0030, *P* = 0.0003), for EIC at 8 months (*P* = 0.0062) and for PS at 1 month (*P* = 0.0023). An interaction effect was seen for diaphragm at 8 and 16 months (*P* = 0.0004, *P* = 0.0448), and for PS at 1 month (*P* = 0.0268). *Post hoc* analyses revealed a significant difference between wild‐type and *mdx* mice in diaphragm at 4 months during baseline (*P* = 0.0003), at 8 months during HcHx (*P* = 0.0003) and at 12 months post vagotomy, HcHx and occlusion (*P* = 0.0108, *P* = 0.0021, *P* = 0.0061), and in EIC at 8 months during baseline and occlusion (*P* = 0.0240, *P* = 0.0408), and in PS at 1 month post vagotomy and occlusion (*P* = 0.0057, *P* = 0.0071), and at 8 months post vagotomy and during HcHx (*P* = 0.0154, *P* = 0.0004).

In summary, despite some changes, on balance there were no major differences between wild‐type and *mdx* mice for mean frequency of respiratory EMG signals.

For power at mean frequency, there was a main effect of time for diaphragm at 1, 8 and 12 months (*P* = 0.0075, *P* = 0443, *P* = 0.0038), for EIC at 1, 4 and 8 months (*P* = 0.0257, *P* = 0029, *P* = 0.0147), and for PS at 1 and 4 months (*P* = 0.0028, *P* = 0.0008) revealing increased respiratory EMG power with increased respiratory demand. There was no genotype effect for diaphragm across all ages. There was a genotype effect for EIC at 1 and 16 months (*P* = 0.0257, *P* = 0.0089), and for PS at 8, 12 and 16 months (*P* = 0.0199, *P* = 0.0071, *P* = 0.0131). An interaction effect was seen for diaphragm at 4, 8 and 12 months (*P* = 0.0008, *P* = 0.0014, *P* = 0.0068), for EIC at 8 months (*P* = 0.0024) and for PS at 1 month (*P* = 0.0038). *Post hoc* analyses revealed a significant difference between wild‐type and *mdx* mice in EIC at 1 month during occlusion (*P* = 0.0111), and at 16 months during occlusion (*P* = 0.0094), and in PS at 16 months of age post vagotomy (*P* = 0.0457).

In summary, despite some changes, on balance there were no major differences between wild‐type and *mdx* mice for power at mean frequency of respiratory EMG signals.

For median frequency, there was a main effect of time (*P <* 0.0001) across all age groups for all three muscles consistent with recruitment of the respiratory muscles during increased ventilatory demand. There was a genotype effect for diaphragm at 1, 4, 8 and 12 months (*P* = 0.0462, *P* = 0.0305, *P* = 0.0018, *P* = 0.0003), for EIC at 8 months (*P* = 0.0068), and for PS at 1 and 8 months (*P* = 0.0012, *P* = 0.0006). An interaction effect was seen for diaphragm at 8 months (*P* = 0.0018) and for PS at 1 month (*P* = 0.0009). *Post hoc* analyses revealed a significant difference between wild‐type and *mdx* mice in diaphragm at 4 months during baseline (*P* = 0.0022), at 8 months during HcHx (*P* = 0.0003), and at 12 months post vagotomy, during HcHx and occlusion (*P* = 0.0323, *P* = 0.0030, *P* = 0.0024), and for PS at 1 month during HcHx and occlusion (*P* = 0.0447, *P* = 0.0097) and at 8 months post vagotomy and during HcHx (*P* = 0.0139, *P* = 0.0008).

In summary, despite some changes, on balance there were no major differences between wild‐type and *mdx* mice for median frequency of respiratory EMG signals.

For power at median frequency, there was a main effect of time for diaphragm at 1 and 8 months (*P* = 0.0071, *P* = 0.0076), for EIC at 1, 4, 8 and 12 months (*P* = 0.0175, *P <* 0.0001, *P* = 0.0219, *P <* 0.0001), and for PS at 1, 4 and 16 months (*P* = 0.0017, *P <* 0.0001, *P* = 0.0072). There was a main genotype effect for EIC at 8 and 16 months (*V* = 0.0482, *P* = 0.0278), and for PS at 1, 8 and 12 months (*P* = 0.0464, *P* = 0.0248, *P* = 0.0084). An interaction effect was seen for diaphragm at 8 months (*P <* 0.0001), for EIC at 8 months (*P <* 0.0001), and for PS at 1 and 8 months (*P* = 0.0393, *P* = 0.0143). *Post hoc* analyses revealed a significant difference between wild‐type and *mdx* mice in EIC at 8 months during baseline (*P <* 0.0001) and in PS at 8 months during occlusion (*P* = 0.0117).

In summary, despite some changes, on balance there were no major differences between wild‐type and *mdx* mice for power at median frequency of respiratory EMG signals.

For diaphragm maximum frequency, there was a main time effect for diaphragm at 1, 4, 8 and 12 months (*P* = 0.0492, *P* = 0.0038, *P* = 0.0423, *P* = 0.0036), for EIC at 1, 4, 8 and 12 months (*P* = 0.0002, *P* = 0.0226, *P* = 0.0003, *P* = 0.0007), and for PS 1, 4, 8 and 16 months (*P* = 0.0096, *P* = 0.0021, *P* = 0.0079, *P* = 0.0053) consistent with respiratory muscle recruitment during increased ventilatory demand. There was a main genotype effect for diaphragm at 4, 8 and 12 months (*P* = 0.0019, *P* = 0.0271, *P* = 0.0018), for EIC at 8 months (*P* = 0.0025), and for PS at 8 months (*P* = 0.0006). An interaction effect was seen for diaphragm at 1, 4, 8 and 12 months (*P* = 0.0059, *P <* 0.0001, *P* = 0.0005, *P <* 0.0001), for EIC at 8 and 16 months (*P* = 0.0001, *P* = 0.0468), and for PS at 4 and 8 months (*P* = 0.0006, *P* = 0.0194). *Post hoc* analyses revealed a significant difference between wild‐type and *mdx* mice in diaphragm at 4 months during baseline (*P* = 0.0152) and at 12 months during HcHx and during occlusion (*P* = 0.0020, *P* = 0.0134), in EIC at 8 months during baseline and occlusion (*P* = 0.0278, *P* = 0.0045), and in PS at 8 months post vagotomy, during HcHx and occlusion (*P* = 0.0004, *P <* 0.0001, *P* = 0.0481).

In summary, despite some changes, on balance there were no major differences between wild‐type and *mdx* mice for maximum frequency of respiratory EMG signals.

For power at maximum frequency, there was a main time effect for EIC at 4 and 12 months (*P* = 0.0010, *P* = 0.0251) and PS at 1 and 16 months (*P* = 0.0008, *P* = 0.0112). There was a main genotype effect for diaphragm at 16 months (*P* = 0.0198), for EIC at 1 and 16 months (*P* = 0.0019, *P* = 0.0209), and for PS at 8 and 12 months (*P* = 0.0164, *P* = 0.0036). An interaction effect was seen for diaphragm, EIC and PS at 8 months (*P <* 0.0001, *P* = 0.0216, *P* = 0.0147). *Post hoc* analyses revealed a significant difference between wild‐type and *mdx* mice in EIC at 1 month during occlusion (*P* = 0.0451), and in PS at 8 months during baseline and occlusion (*P* = 0.0179, *P* = 0.0240), and at 12 months during baseline (*P* = 0.0256).

In summary, despite some changes, on balance there were no major differences between wild‐type and *mdx* mice for power at maximum frequency of respiratory EMG signals.

For diaphragm total power (AUC of the frequency spectrum), there was a main time effect for diaphragm at 1, 4, 8 and 12 months (*P* = 0.0140, *P* = 0.0061, *P* = 0.0022, *P* = 0.0348), for EIC at 1 and 8 months (*P* = 0.0195, *P* = 0.0006), and for PS at 4 and 8 months (*P* = 0.0002, *P* = 0.0022). There was a main genotype effect for diaphragm at 4, 8 and 16 months (*P* = 0.0456, *P* = 0.0197, *P* = 0.0094), for EIC at 1, 12 and 16 months (*P* = 0.0319, *P* = 0.0153, *P* = 0.0172), and for PS at 8 and 12 months (*P* = 0.0029, *P* = 0.0015). An interaction effect was seen for diaphragm at 4, 8 and 12 months (*P* = 0.0038, *P* = 0.0125, *P* = 0.0032), for EIC at 1, 8 and 16 months (*P* = 0.0164, *P* = 0.0116, *P* = 0.0494), and for PS at 1 and 8 months (*P* = 0.0105, *P* = 0.0009). *Post hoc* analyses revealed a significant difference between wild‐type and *mdx* mice in diaphragm at 4 and 8 months during occlusion (*P* = 0.0437, *P* = 0.0430), in EIC at 1 month during occlusion (*P* = 0.0165), and at 16 months during baseline and occlusion (*P* = 0.0475, *P* = 0.0252), and in PS at 8 months during baseline, post vagotomy and occlusion (*P* = 0.0170, *P* = 0.0148, *P* = 0.0041), and at 12 month during baseline (*P* = 0.0244).

In summary, despite some changes, on balance there were no major differences between wild‐type and *mdx* mice for total power of respiratory EMG signals.

### 3.2 Neuromechanical efficiency

**FIGURE 4:**
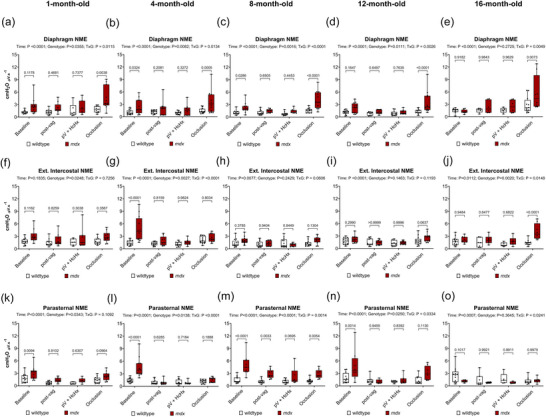
Respiratory muscle neuromechanical efficiency (NME) during baseline, following bilateral vagotomy, during subsequent exposure to hypercapnic hypoxia (HcHx) and during sustained tracheal occlusion in anaesthetised wild‐type and *mdx* mice. Group data for diaphragm (a–e), external intercostal (f–j) and parasternal intercostal (k–o) NME during baseline, post‐bilateral vagotomy, subsequent exposure to HcHx and during sustained tracheal occlusion in wild‐type and *mdx* mice at 1, 4, 8, 12 and 16 months of age. Values are expressed as box (median, IQR and individual data points) and whisker (min to max) plots. Data were statistically compared by two‐way mixed ANOVA with Šidák's multiple comparisons *post hoc* test. Absolute *P*‐values for all comparisons are reported.

Figure 4 shows inspiratory pressure and respiratory EMG data represented as NME in anaesthetised wild‐type and *mdx* mice during baseline, following bilateral vagotomy, during subsequent exposure to HcHx and finally peak responses during sustained tracheal occlusion. NME for diaphragm (Figure 4a–e), EIC (Figure 4f–j) and PS (Figure 4k–o) are shown.

For NME there was a main time effect for diaphragm across all age groups (*P <* 0.0001), for EIC at 4, 8, 12 and 16 months (*P <* 0.0001, *P* = 0.0077, *P <* 0.0001, *P* = 0.0112), and for PS across all age groups (*P <* 0.0001, *P <* 0.0001, *P <* 0.0001, *P <* 0.0001, *P* = 0.0007) revealing expected changes associated with increased ventilatory demand. There was a main genotype effect for diaphragm at 1, 4, 8 and 12 months (*P* = 0.0355, *P* = 0.0082, *P* = 0.0016, *P* = 0.0111), for EIC at 1, 4 and 16 months (*P* = 0.0248, *P* = 0.0027, *P* = 0.0020), and for PS at 1, 4, 8 and 12 months (*P* = 0.0343, *P* = 0.0138, *P <* 0.0001, *P* = 0.0250). An interaction effect was seen for diaphragm at all age groups (*P* = 0.0115, *P* = 0.0134, *P <* 0.0001, *P* = 0.0026, *P* = 0.0049), for EIC at 4 and 16 months (*P <* 0.0001, *P* = 0.0148), and for PS at 4, 8, 12 and 16 months (*P <* 0.0001, *P* = 0.0014, *P* = 0.0334, *P* = 0.0241). *Post hoc* analyses revealed a significant difference between wild‐type and *mdx* mice in diaphragm at 1 month during occlusion (*P* = 0.0038), at 4 months during baseline and occlusion (*P* = 0.0324, *P* = 0.0005), at 8 months of age during baseline and occlusion (*P* = 0.0286, *P <* 0.0001) and at 12 months during occlusion (*P <* 0.0001). There was a significant difference between wild‐type and *mdx* mice for EIC at 4 months during baseline (*P <* 0.0001) and at 16 months during occlusion (*P <* 0.0001). Differences were also noted for PS at 1 month during baseline (*P* = 0.0094), at 4 months during baseline (*P <* 0.0001), at 8 months during baseline, post vagotomy and occlusion (*P <* 0.0001, *P* = 0.0033, *P* = 0.0054), and at 12 months during baseline (*P* = 0.0014).

Apparent increases in NME during progressive dystrophic disease, particularly during peak activation associated with tracheal occlusion, arise due to preservation of pressure‐generating capacity, which we have previously demonstrated is a compensation afforded by accessory muscles of breathing (O'Halloran et al., 2023). This compensation contributes to an apparent increase in efficiency in the obligatory muscles of breathing, that is, maintained pressure‐generation despite decreases in obligatory respiratory EMG activity, but it is unlikely that NME is truly improved in *mdx* obligatory muscles.

#### 3.2.1 Tension–time index

**FIGURE 5:**
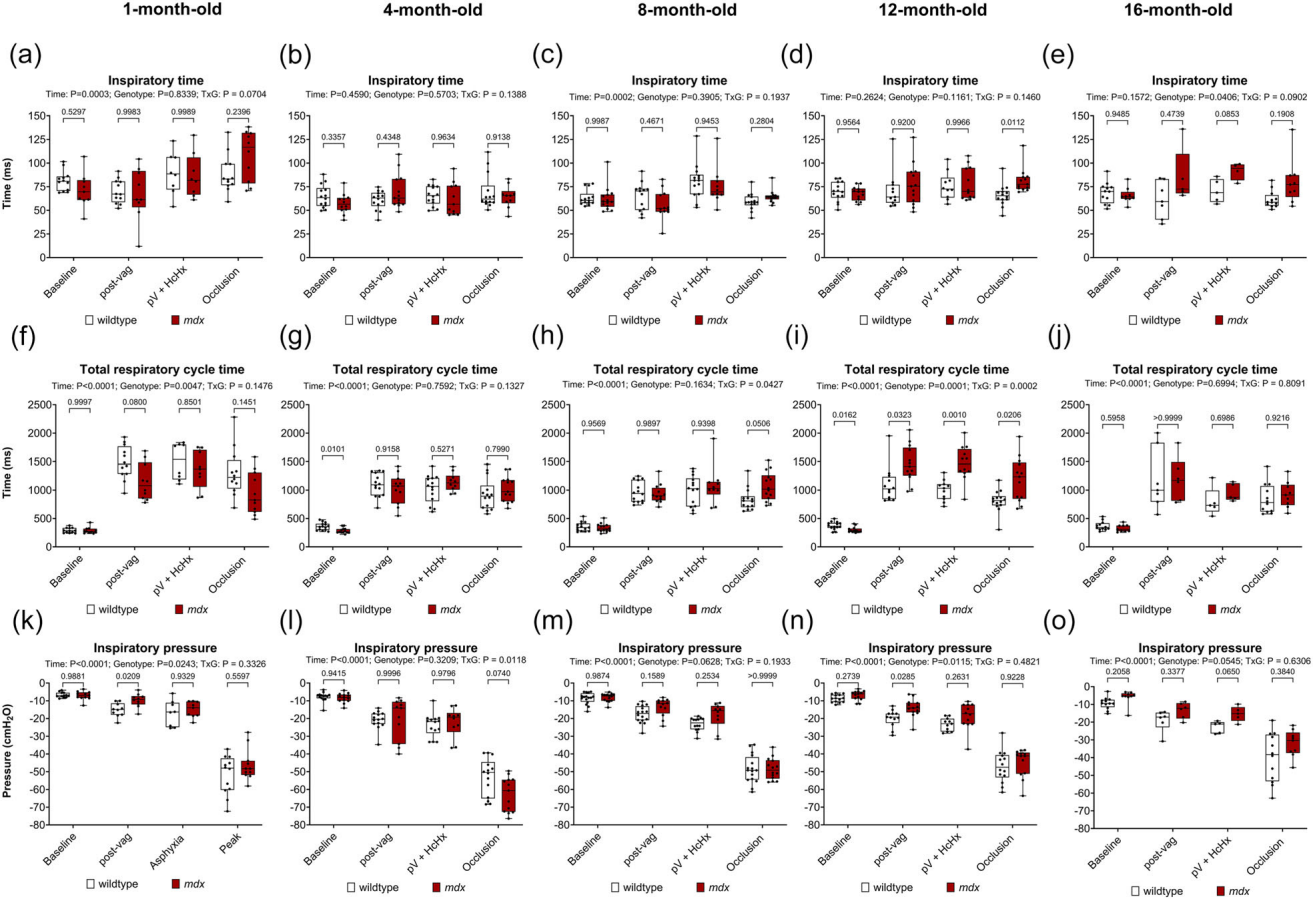
Inspiratory time, total respiratory cycle time and inspiratory pressure during baseline, following bilateral vagotomy, during subsequent exposure to hypercapnic hypoxia (HcHx) and sustained tracheal occlusion in anaesthetised wild‐type and *mdx* mice. Group data for inspiratory time (a–e), total respiratory cycle time (f–j) and inspiratory pressure (k–o) during baseline, post‐bilateral vagotomy, subsequent exposure to HcHx and sustained tracheal occlusion in wild‐type and *mdx* mice at 1, 4, 8, 12 and 16 months of age. Values are expressed as box (median, IQR and individual data points) and whisker (min to max) plots. Data were statistically compared by mixed affect analysis with Šidák's multiple comparisons *post hoc* test. Absolute *P*‐values for all comparisons are reported.

Figure 5 shows inspiratory time (Figure 5a–e), total respiratory cycle time (Figure 5f–j) and inspiratory pressure (Figure 5k–o) data in anaesthetised wild‐type and *mdx* mice during baseline, following bilateral vagotomy, during subsequent exposure to HcHx and finally for peak responses obtained during sustained tracheal occlusion.

For inspiratory time there was a main time effect at 1 and 8 months (*P* = 0.0003, *P* = 0.0002). There was a main genotype effect at 16 months (*P* = 0.0406) and no interaction effect. *Post hoc* analyses revealed inspiratory time was equivalent between wild‐type and *mdx* mice.

For total respiratory cycle time there was a main time effect at all ages (*P <* 0.0001), a main genotype effect at 1 and 12 months (*P* = 0.0047, *P* = 0.0001), and an interaction effect at 8 and 12 months (*V* = 0.0427, *P* = 0.00020). *Post hoc* analyses revealed a significant difference between wild‐type and *mdx* mice at 12 months during baseline, post vagotomy, HcHx and occlusion (*P* = 0.0162, *P* = 0.0323, *P* = 0.0010, *P* = 0.0206) (Figure 5I).

For inspiratory pressure there was a main time effect at all ages (*P <* 0.0001), a genotype effect at 1 and 12 months (*P* = 0.0243, *P* = 0.0115) and an interaction effect at 4 months (*P* = 0.0118). *Post hoc* analyses revealed a significant difference between wild‐type and *mdx* mice at 1 and 12 months post vagotomy (*P* = 0.0209, *P* = 0.0285).

**FIGURE 6:**
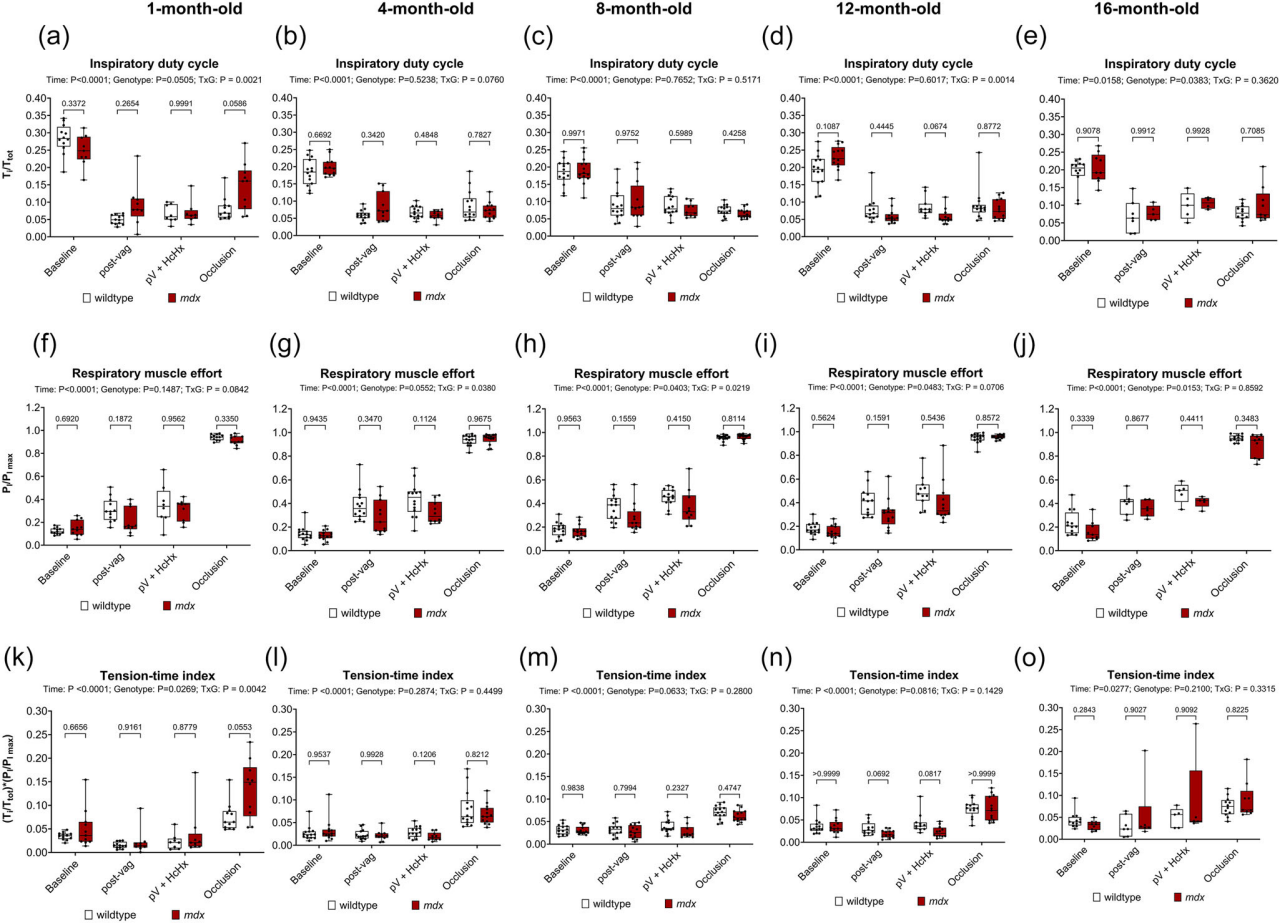
Inspiratory duty cycle, respiratory effort, and tension–time index (TTI) during baseline, post‐bilateral vagotomy, during subsequent exposure to hypercapnic hypoxia (HcHx), and finally peak responses during sustained tracheal occlusion in anaesthetised wild‐type and *mdx* mice. Group data for inspiratory duty cycle (a–e), respiratory muscle effort (f–j) and TTI (k–o) during baseline, post‐bilateral vagotomy, during subsequent exposure to HcHx, and finally peak responses during sustained tracheal occlusion in wild‐type and *mdx* mice at 1, 4, 8, 12 and 16 months of age. Values are expressed as box (median, 25–75 percentile and individual data points) and whisker (min to max) plots. Data were statistically compared by two‐way mixed ANOVA with Šidák's multiple comparisons *post hoc* test. Absolute *P*‐values for all *post hoc* comparisons are reported.

Figure 6 shows the parameters that determine the TTI, which include inspiratory time divided by the total respiratory cycle time (i.e. inspiratory duty cycle) (Figure 6a–e), inspiratory pressure divided by the maximum inspiratory pressure (i.e. respiratory muscle effort) (Figure 6f–j), and the product of these two ratios which is TTI (Figure 6k–o). For inspiratory duty cycle there was a main time effect for all age groups (*P <* 0.0001, *P <* 0.0001, *P <* 0.0001, *P <* 0.0001, *P* = 0.0158), a genotype effect at 16 months (*P* = 0.0383), and an interaction effect at 1 and 12 months (*P* = 0.0021, 0.0014). *Post hoc* analyses revealed no significant differences between wild‐type and *mdx* for inspiratory duty cycle.

For respiratory muscle effort there was a main time effect across all age groups (*P <* 0.0001), a genotype effect at 8, 12 and 16 months (*P* = 0.0403, *P* = 0.0483, *P* = 0.0153), and an interaction effect at 4 and 8 months (*P* = 0.0380, *P* = 0.0219). *Post hoc* analyses revealed no significant differences between wild‐type and *mdx* for respiratory muscle effort.

For TTI there was a time effect for all age groups (*P <* 0.0001, *P <* 0.0001, *P <* 0.0001, *P <* 0.0001, *P* = 0.0277), a genotype effect at 1 month (*P* = 0.0269), and an interaction effect at 1 month (*P* = 0.0042). *Post hoc* analyses revealed no significant differences between wild‐type and *mdx* for TTI.

In summary, despite some changes, there were no major differences between wild‐type and *mdx* mice for TTI across behaviours.

### 3.3 Inspiratory drive rate and EMG rise time

**FIGURE 7:**
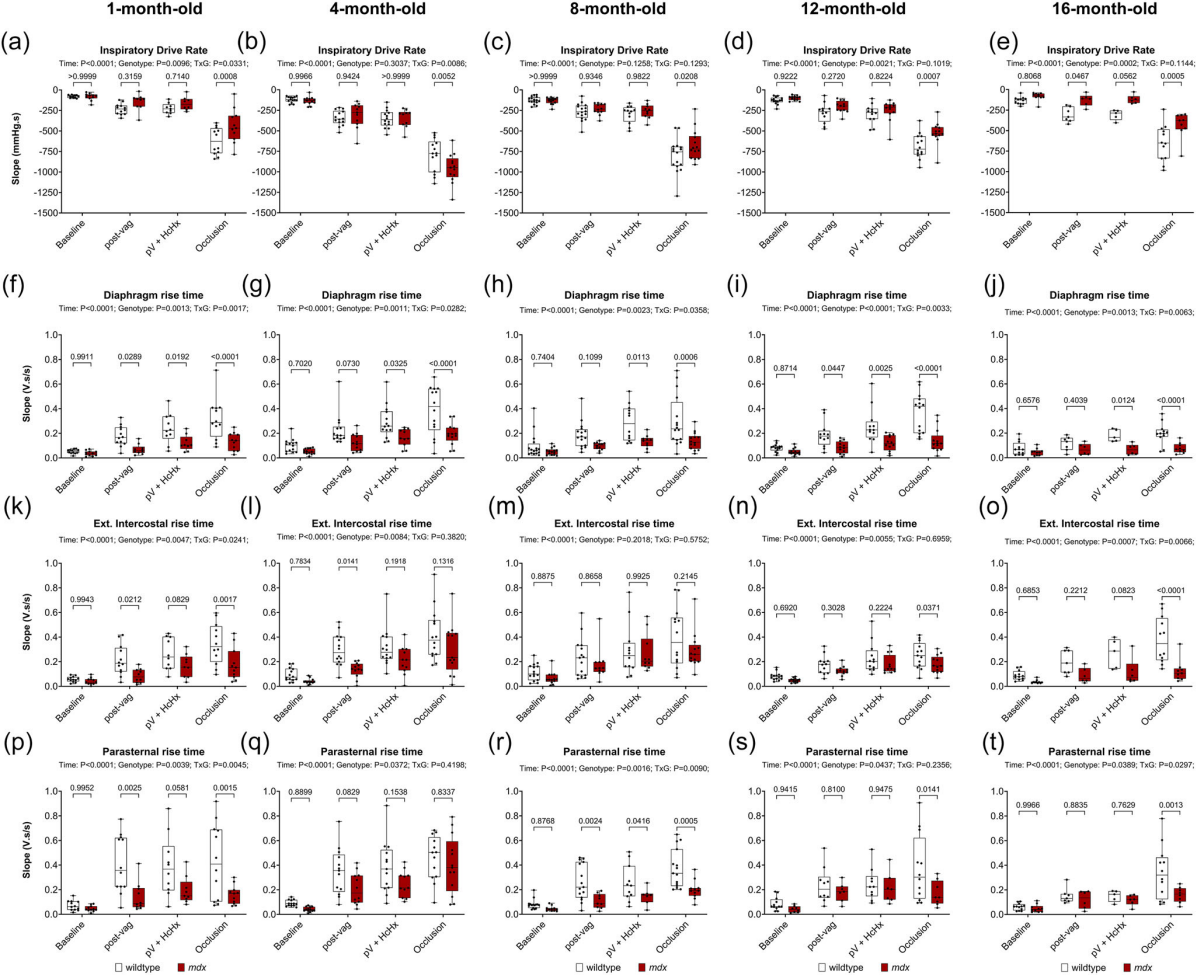
Slope of inspiratory pressure, and diaphragm, EIC and PS integrated EMG recordings during baseline, post‐bilateral vagotomy, during subsequent exposure to hypercapnic hypoxia (HcHx), and peak responses during sustained tracheal occlusion in anaesthetised wild‐type and *mdx* mice. Group data for slope of inspiratory pressure (a–e), integrated diaphragm EMG (f–j), integrated EIC EMG (k–o), and integrated PS EMG (p–t), during baseline, post‐bilateral vagotomy, during subsequent exposure to HcHx, and finally peak responses during sustained tracheal occlusion in wild‐type and *mdx* mice at 1, 4, 8, 12 and 16 months of age. Values are expressed as box (median, 25–75 percentile and individual data points) and whisker (min to max) plots. Data were statistically compared by two‐way mixed ANOVA with Šidák's multiple comparisons *post hoc* test. Absolute *P*‐values for all *post hoc* comparisons are reported.

Figure 7 shows the slope of inspiratory pressure (Figure 7a–e), integrated diaphragm EMG (Figure 7f–j), integrated EIC EMG (Figure 7k–o), and integrated PS EMG (Figure 7p–t) data in anaesthetised wild‐type and *mdx* mice during baseline, following bilateral vagotomy, during subsequent exposure to HcHx and for peak responses obtained during sustained tracheal occlusion.

For inspiratory drive rate (IDR) there was a main time effect for all age groups (*P <* 0.0001), a genotype effect at 1, 12 and 16 months (*P* = 0.0096, *P* = 0.0021, *P* = 0.0002), and an interaction effect at 1 and 4 months (*P* = 0.0331, *P* = 0.0086). *Post hoc* analyses revealed a significant difference between wild‐type and *mdx* during occlusion at 1, 12 and 16 months (*P* = 0.0008, *P* = 0.0007, *P* = 0.0005) (Figure 7a–e).

In summary, IDR is lower in *mdx* mice compared to wild‐type mice from early to advanced disease reflecting impaired neuromuscular function.

For diaphragm EMG rise time there was a main time effect across all ages (*P <* 0.0001), a main genotype effect across all ages (*P* = 0.0013, *P* = 0.0011, *P* = 0.0023, *P <* 0.0001, *P* = 0.0013) and an interaction effect across all ages (*P* = 0.0017, *P* = 0.0282, *P* = 0.0023, *P* = 0.0033, *P* = 0.0063). *Post hoc* analyses revealed a significant difference between wild‐type and *mdx* at 1 month post vagotomy, HcHx and occlusion (*P* = 0.0289, *P* = 0.0192, *P <* 0.0001), at 4 months during HcHx and occlusion (*P* = 0.0325, *P <* 0.0001), at 8 months during HcHx and occlusion (*P* = 0.0113, *P* = 0.0006), at 12 months post vagotomy, HcHx and occlusion (*P* = 0.0447, *P* = 0.0025, *P <* 0.0001), and at 16 months during HcHx and occlusion (*P* = 0.0124, *P <* 0.0001) (Figure 7f–j).

For EIC EMG rise time there was a main time effect across all ages (*P <* 0.0001), a main genotype effect at 1, 4, 12 and 16 months (*P* = 0.0047, *P* = 0.0084, *P* = 0.0055, *P* = 0.0007), and an interaction effect at 1 and 16 months of age (*P* = 0.0241, *P* = 0.0066). *Post hoc* analyses revealed a significant difference between wild‐type and *mdx* at 1 month of age post vagotomy and occlusion (*P* = 0.0212, *P* = 0.0017), at 4 months post vagotomy (*P* = 0.0141), at 12 months during occlusion (*P* = 0.0371), and at 16 months during occlusion (*P <* 0.0001) (Figure 7k–o).

For PS EMG rise time there was a main time effect across all ages (*P <* 0.0001), a main genotype effect at all ages (*P* = 0.0039, *P* = 0.0372, *P* = 0.0016, *P* = 0.0437, *P* = 0.0389), and an interaction effect at 1, 8 and 16 months of age (*P* = 0.0045, *P* = 0.0090, *P* = 0.0297). *Post hoc* analyses revealed a significant difference between wild‐type and *mdx* at 1 month post vagotomy and occlusion (*P* = 0.0025, *P* = 0.0015), at 8 months post vagotomy, HcHx and occlusion (*P* = 0.0024, *P* = 0.0416, *P* = 0.0005), at 12 months during occlusion (*P* = 0.0141), and 16 months during occlusion (*P* = 0.0013) (Figure 7p–t).

In summary, there was a tendency for lower respiratory EMG rise times in *mdx* mice compared with wild‐type mice, but the effects were not consistent across all behaviours.

### 3.4 Temporal pressure–time relationship during sustained tracheal occlusion from onset until task failure

**FIGURE 8:**
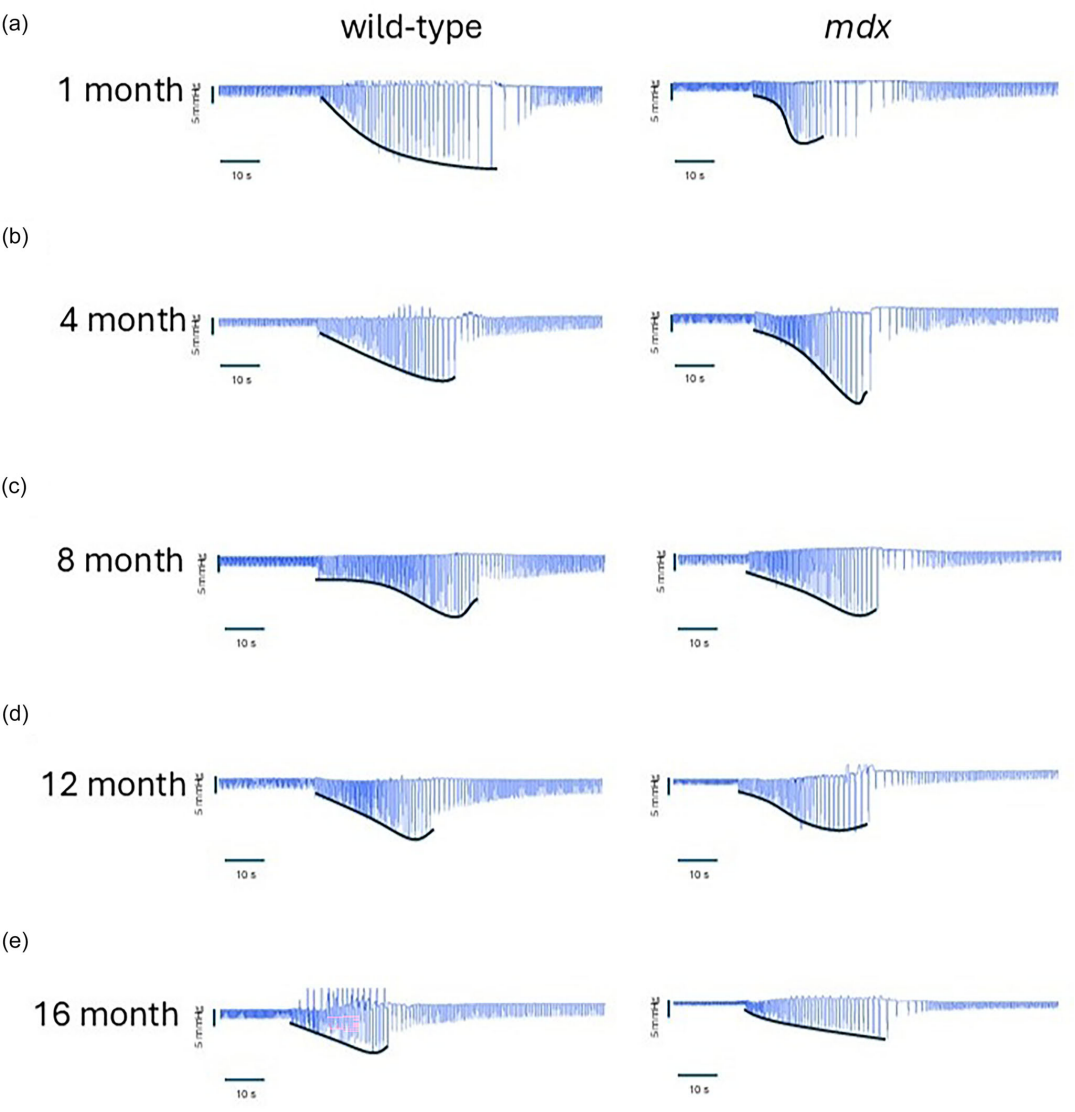
Original traces of inspiratory pressure during protracted airway occlusion for wild‐type and *mdx* at 1 (a), 4 (b), 8 (c), 12 (d), and 16 (e) months of age. Black lines represent the pressure–time plots that were generated from individual pressures generated and of which AUC was generated.

Original inspiratory pressure recordings are shown across all behaviours in Figure 8. The black ‘envelope’ lines on the original traces represent the pressure–time relationship during sustained tracheal occlusion from onset until task failure.

**FIGURE 9:**
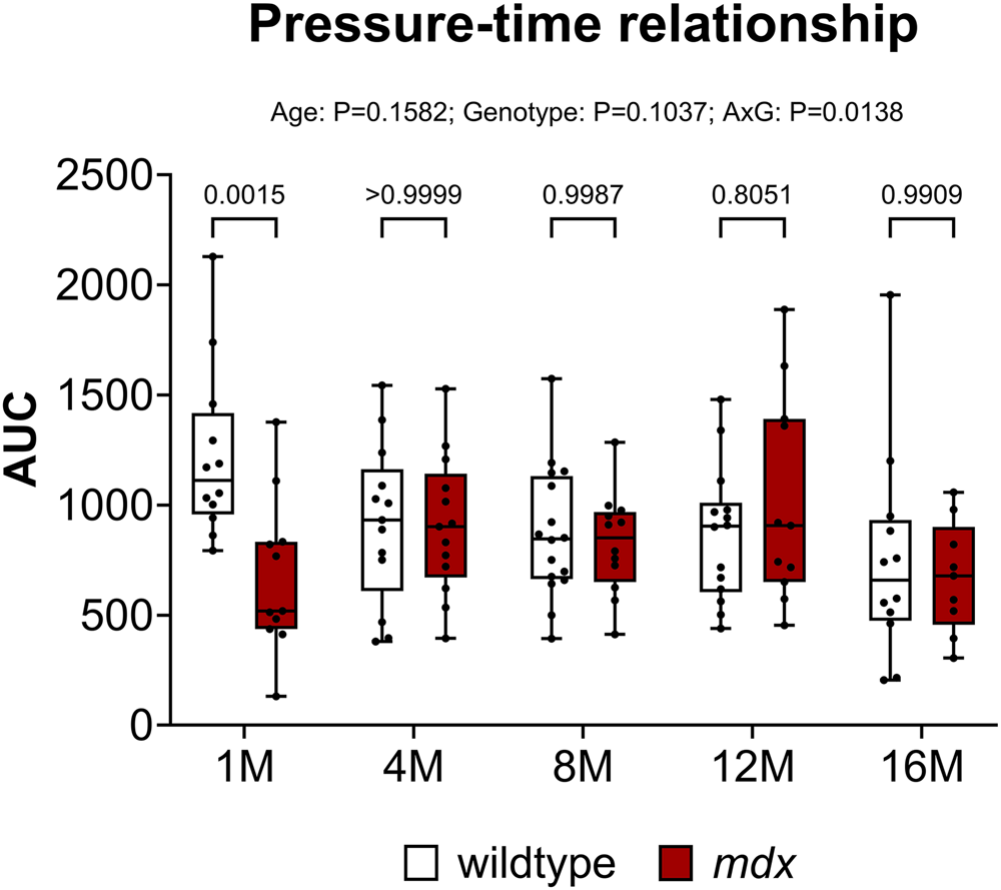
Pressure–time relationship during sustained tracheal occlusion from onset until task failure in anaesthetised wild‐type and *mdx* mice. Group data for the area under the curve of the pressure–time relationship during a sustained tracheal occlusion in wild‐type and *mdx* mice at 1, 4, 8, 12 and 16 months of age. Values are expressed as box (median, IQR and individual data points) and whisker (min to max) plots. Data were statistically compared by mixed two‐way ANOVA analysis with Šidák's multiple comparisons *post hoc* test. Absolute *P*‐values for all comparisons are reported.

Figure 9 shows data for area under the curve (AUC) of the pressure–time relationship determined during a single sustained tracheal occlusion from the first effort until task failure in anaesthetised wild‐type and *mdx* mice. For pressure–time AUC there was no main age effect (*P* = 0.1582), no main genotype effect (*P* = 0.1037), but an interaction effect (*P* = 0.0138).

In summary, the composite pressure–time relationship was altered in *mdx* mice at 1 month of age revealing impaired pressure‐generating capacity (associated with known diaphragm weakness), but thereafter the relationship was compensated and unaltered between wild‐type and *mdx* mice even in advanced disease at 16 months of age.

### 3.5 Entropy‐based signal complexity measures

**FIGURE 10:**
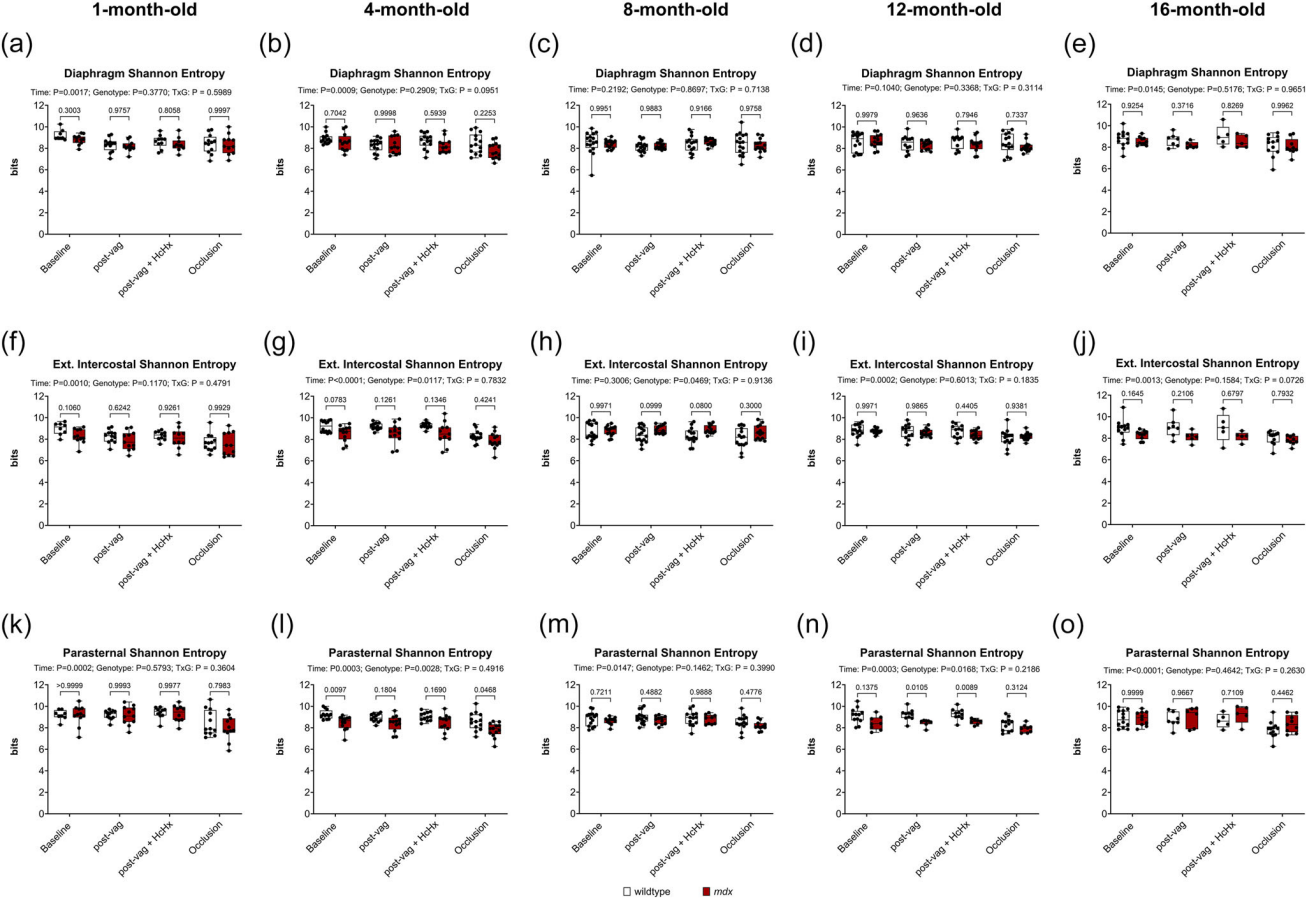
Shannon entropy of respiratory muscle EMG signals in anaesthetised wild‐type and *mdx* mice. Group data for Shannon entropy (bits) measured from diaphragm (a–e), external intercostal (f–j), and parasternal intercostal (k–o) muscle EMG signals during baseline, post‐vagotomy, hypercapnic hypoxia (HcHx) and sustained tracheal occlusion. Values are expressed as box (median, IQR and individual data points) and whisker (min to max) plots. Data were statistically compared by mixed two‐way ANOVA analysis with Šidák's multiple comparisons *post hoc* test. Absolute *P*‐values for all comparisons are reported.

Figure 10 shows Shannon entropy, representing the distribution in amplitude of EMG signals in Diaphragm (Figure 10a–e), EIC (Figure 10f–j) and PS (Figure 10k–o) in anaesthetised wild‐type and *mdx* mice during baseline, following bilateral vagotomy, during subsequent exposure to HcHx and for peak responses obtained during sustained tracheal occlusion. For Diaphragm there was a main time effect at 1, 4 and 16 months (*P* = 0.0017, *P* = 0.0009, *P* = 0.0145), no genotype effect, and no interaction effect across all age groups. For EIC there was a main time effect at 1, 4, 12 and 16 months (*P* = 0.0010, *P <* 0.0001, *P* = 0.0002, *P* = 0.0013), a main genotype effect at 4 and 8 months (*P* = 0.0117, *P* = 0.0469) (Figure 10g, h), and no interaction effect across all age groups. For PS there was a main time effect for all age groups (*P* = 0.0002, *P* = 0.0003, *P* = 0.0147, *P* = 0.0003, *P <* 0.0001), a main genotype effect at 4 and 12 months (*P* = 0.0028, *P* = 0.0168) (Figure 10l, n), and no interaction effect across all age groups. *Post hoc* analyses revealed a significant difference between wild‐type and *mdx* at 4 months of during baseline and occlusion (*P* = 0.0097, *P* = 0.0468), and at 12 months post vagotomy (*P* = 0.0105) and HcHx (*P* = 0.0089) (Figure 10k–o).

**FIGURE 11:**
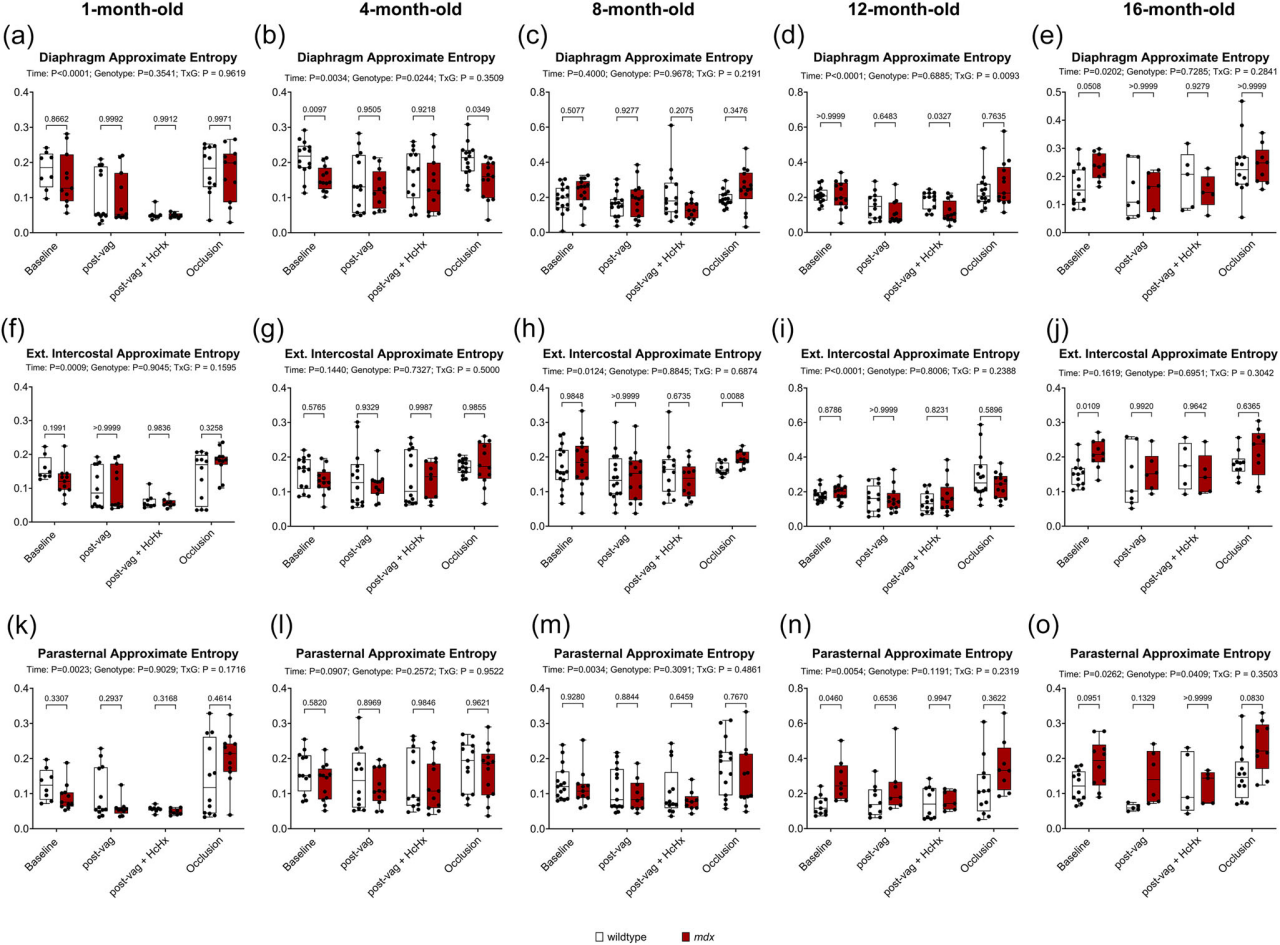
Approximate entropy of respiratory muscle EMG signals in anaesthetised wild‐type and *mdx* mice. Group data for approximate entropy measured from diaphragm (a–e), external intercostal (f–j), and parasternal intercostal (k–o) muscle EMG signals during baseline, post‐vagotomy, hypercapnic hypoxia (HcHx) and sustained tracheal occlusion. Values are expressed as box (median, IQR and individual data points) and whisker (min to max) plots. Data were statistically compared by mixed two‐way ANOVA analysis with Šidák's multiple comparisons *post hoc* test. Absolute *P*‐values for all comparisons are reported.

Figure 11 shows approximate entropy in Diaphragm (Figure 11a–e), EIC (Figure 11f–j), and PS (Figure 11k–o) in anaesthetised wild‐type and *mdx* mice during baseline, following bilateral vagotomy, during subsequent exposure to HcHx and for peak responses obtained during sustained tracheal occlusion. For Diaphragm there was a main time effect at 1, 4, 12 and 16 months (*P <* 0.0001, *P* = 0.0034, *P <* 0.0001, *P* = 0.0202), a main genotype effect at 4 months (*p* = 0.0244), and an interaction effect at 12 months (*P* = 0.0093). *Post hoc* analyses revealed a significant difference between wild‐type and *mdx* at 4 months during baseline and occlusion (*P* = 0.0097, *P* = 0.0349) (Figure 11b). For EIC there was a main time effect at 1, 8 and 12 months (*P* = 0.0009, *P* = 0.0124, *P <* 0.0001), no main genotype effect, and no interaction effect across all age groups. For PS there was a main time effect at 1, 8, 12 and 16 months (*P* = 0.0023, *P* = 0.0034, *P* = 0.0054, *P* = 0.0262), a main genotype effect 16 months (*P* = 0.0409), and no interaction effect across all age groups.

**FIGURE 12:**
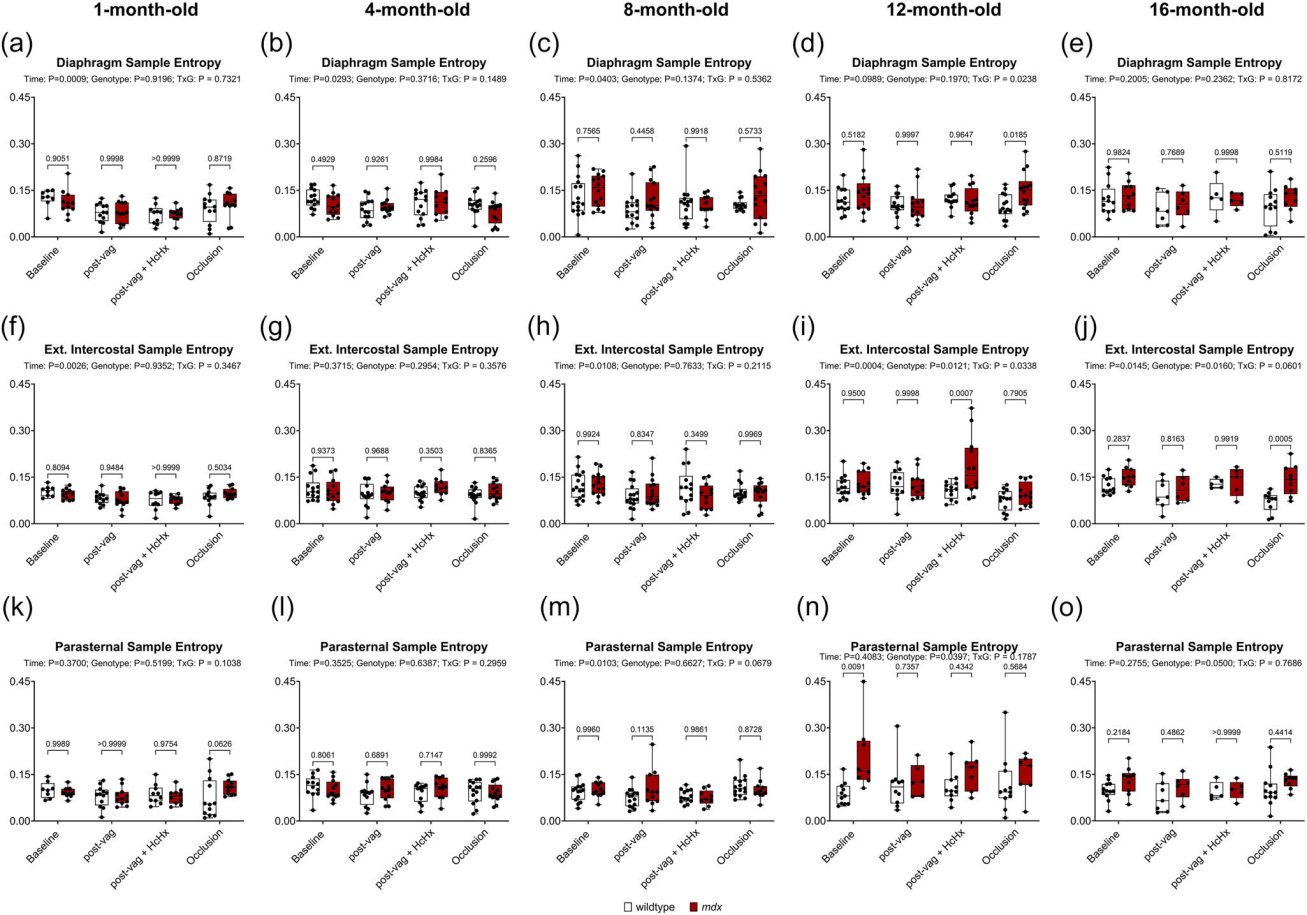
Sample entropy of respiratory muscle EMG signals in anaesthetised wild‐type and *mdx* mice. Group data for sample entropy measured from diaphragm (a–e), external intercostal (f–j), and parasternal intercostal (k–o) muscle EMG signals during baseline, post‐vagotomy, hypercapnic hypoxia (HcHx) and sustained tracheal occlusion. Values are expressed as box (median, IQR and individual data points) and whisker (min to max) plots. Data were statistically compared by mixed two‐way ANOVA analysis with Šidák's multiple comparisons *post hoc* test. Absolute *P*‐values for all comparisons are reported.

Figure 12 shows sample entropy in diaphragm (Figure 12a–e), EIC (Figure 12f–j) and PS (Figure 12k–o) in anaesthetised wild‐type and *mdx* mice during baseline, following bilateral vagotomy, during subsequent exposure to HcHx and for peak responses obtained during sustained tracheal occlusion. For diaphragm there was a main time effect at 1, 4 and 8 months (*P* = 0.0009, *P* = 0.0293, *P* = 0.0403), no main genotype effect, and an interaction effect at 12 months (*P* = 0.0238). For EIC there was a main time effect at 1, 8, 12 and 16 months (*P* = 0.0026, *P* = 0.0108, *P* = 0.0004, *P* = 0.0145), a main genotype effect at 12 and 16 months (*P* = 0.0121, *P* = 0.0160), and an interaction effect at 12 months (*P* = 0.0338). *Post hoc* analyses revealed a significant difference between wild‐type and *mdx* at 12 months during HcHx (*P* = 0.0007) (Figure 12i), and at 16 months during occlusion (*P* = 0.0005) (Figure 12j). For PS there was a main time effect at 8 months (*P* = 0.0103), a main genotype effect at 12 months (*P* = 0.0397) and no interaction effect across all age groups. *Post hoc* analyses revealed a significant difference between wild‐type and *mdx* at 12 months during baseline (*P* = 0.0091) (Figure 12n).

In summary, although some differences were noted, for the most part entropy measures of respiratory EMG signals were equivalent between wild‐type and *mdx* mice across the natural history of disease.

## 4 DISCUSSION

A comprehensive understanding of the respiratory phenotype across the natural history of disease in animal models of DMD is required to facilitate translational research focussed on novel therapeutics for respiratory insufficiency. The *mdx* mouse exhibits a distinct respiratory pathophysiology characterised by early‐onset respiratory muscle histopathology and weakness, as well as EMG deficits in primary inspiratory muscles, which persist and progress throughout the lifespan (O'Halloran et al., 2023; Slyne et al., 2025). The current study was motivated by the need to expand upon characterization of the respiratory phenotype in *mdx* mice to consider the extent to which the mouse model provides a platform for interventional studies. We focused on clinically relevant measures to characterise the potential utility of the *mdx* mouse as a translatable pre‐clinical model of respiratory system dysfunction in DMD.

MIP is an important measure of respiratory muscle strength. Point measures of MIP are useful indirect indices of the peak strength of inspiratory musculature. However, we also acknowledge that volitional tests have limitations in the assessment of people with DMD as they may be unable to perform the manoeuvres correctly due to age or understanding, highly limiting its clinical applicability (Fauroux et al., 2015; Hinton et al., 2007; Khirani et al., 2014).

The trajectory of MIP across the natural history of disease in *mdx* mice demonstrates a paradoxical recovery phase in young *mdx* mice before progressive late‐stage decline (O'Halloran et al., 2023; Slyne et al., 2025). In the present study, the pressure–time relationship during sustained occlusion was reduced in *mdx* mice at 1 month of age consistent with diaphragm weakness. However, this composite assessment of pressure‐generating capacity was thereafter compensated and equivalent to wild‐type through 16 months of age, indicating preserved capacity to sustain high demand respiratory work owing to sustained compensation by accessory muscles of breathing (O'Halloran et et al., 2023; Slyne et al., 2025).

NRD is used to assess the mechanical load on the respiratory muscles (Cavalcante et al., 2024; Jolley et al., 2009; Murphy et al., 2011; Onofri et al., 2018; Reilly et al., 2016, 2011; Steier et al., 2017). Peak respiratory EMG activity is decreased in *mdx* mice, presenting in early disease, which complicates the use and interpretation of NRD since the reference maximum values for respiratory EMG activities are lower in *mdx* mice. Nevertheless, NRD was essentially equivalent across behaviours between wild‐type and *mdx* mice with similar sequential increases in NRD with increased ventilatory demand suggesting preserved mechanisms of reflex motor recruitment of the respiratory musculature in *mdx* mice. The absolute levels of electrical activation, that is, composite motor unit activity, in the respiratory EMGs are lower in *mdx* mice, suggesting impaired neural activation of muscle, which could relate to axonopathy, which has been described in respiratory motor nerves in 12‐month‐old *mdx* mice (Dhindsa et al., 2020), and/or impaired neuromuscular transmission (Personius & Sawyer, 2006). Moreover, inspiratory drive rate derived from inspiratory pressure recordings was lower in *mdx* mice during maximal activation of the respiratory system during sustained tracheal occlusion, an effect seen across the natural history of the disease suggesting deficits in maximal central respiratory drive. Sixteen‐month‐old *mdx* mice show an increased baseline NRD, and although this is suggestive of a greater mechanical load necessitating a greater drive to breathe at baseline in *mdx*, this outcome arises due to the major reduction in peak EMG activity in 16‐month‐old *mdx* mice, that is, absolute baseline EMG activity in *mdx* mice is less than wild‐type. NRD has been used clinically to assess the progression of COPD (Jolley et al., 2009; Murphy et al., 2011), obesity hypoventilation syndrome (Onofri et al., 2018), hypertension (Cavalcante et al., 2024) and cystic fibrosis (Reilly et al., 2013, 2011, 2016) but has not been used previously in DMD. Regular NRD testing could ostensibly facilitate progressive disease monitoring and management in DMD but serious limitations in its use are revealed in our study in *mdx* mice, due to the complex nature of neuromuscular disease.

Continuous monitoring of skeletal muscles through intra‐muscular EMG and its component frequency spectrum has been used for fatigue analysis. As a muscle becomes fatigued there is a shift towards lower frequencies of myoelectric signal power spectrum (Cifrek et al., 2009). Muscle fatigue during sustained contraction is characterised by decreases in muscle fibre conduction velocity, which directly changes the shape of the motor unit action potential (Yaar & Niles, 1992). With an increase in intra‐muscular pressure during a certain level of contraction, the muscle becomes ischaemic when blood flow is stopped, and intracellular pH is decreased with the accumulation of metabolites resulting in the decrease in muscle fibre conduction velocity, alteration of the motor unit action potential and a decrease in median frequency (Brody et al., 1991). Reduction of the muscle fibre conduction velocity is one of the causes of signal power spectrum shift towards lower frequencies (Brody et al., 1991). During sustained tracheal occlusion, used in our study to elicit maximum respiratory EMG activation, for the most part signal frequencies were equivalent between wild‐type and *mdx* mice across 1–16 months indicating that despite the significant decrease in *mdx* respiratory EMG amplitudes, spectral features remain intact in the *mdx* mouse.

Similarly, comparisons can be drawn between combined EMG and frequency spectrum analysis and force. A decrease in muscle force can arise due to reduced EMG amplitude, and EMG spectrum shifts to the left (O'Halloran et al., 2023; Hägg et al., 2000).

Pandeya et al. (2023) compared the frequency spectrum of wild‐type and D2.*mdx* mice and showed greater power at higher frequency in the animals with muscular dystrophy and fewer low frequency components, consistent with short duration, polyphasic motor units, characteristic of myopathy, which could be due to asynchronous activation of the damaged fibres. Frascarelli et al. (1988) showed in the biceps branchii of DMD boys that there was a greater amount of high frequency activity which in turn causes a shift in median frequency towards higher values. In the current study, this shift was not seen in the *mdx* diaphragm, EIC and PS muscles, and MNF and MDF were shown to be equivalent indicating that the *mdx* mouse does not display the EMG spectral characteristics observed in the D2.*mdx* model and people with DMD.

Total power was calculated from AUC of the frequency spectrum. Total power has been used in place of the integral of the EMG signal as EMG rectification can alter the power spectrum of recorded EMG signals (Neto & Christou, 2010). Frascarelli et al. (1988) showed that the total power in biceps branchii muscles of DMD boys was lower than in controls. Despite significant reductions in *mdx* diaphragm EMG amplitude during maximal activation of the muscle at all age groups (1–16 months), total power was lower than wild‐type values only at 4 and 8 months of age. Interestingly, several measures of entropy, which assessed the complexity of EMG signals, were similar in wild‐type and *mdx* obligatory muscles across disease progression.

NME measures the efficiency of conversion of neural activation to pressure generation (Bellani et al., 2013; Jansen et al., 2018; Lozano‐Garcia et al., 2019; Lozano‐García et al., 2021). Apparent increases in *mdx* diaphragm NME during occlusion observed in our study most likely reflect accessory muscle contributions to preserved inspiratory pressure generation rather than improved efficiency in the diaphragm per se. Unlike the scenario in *mdx* mice, MIP declines in a linear fashion due to age‐related decline after the peak in respiratory system performance compounded further by severity of disease in DMD (Fauroux et al., 2015; Khirani et al., 2014). The complexities of accessory muscle compensation in the *mdx* mouse model obscure the potential utility of diaphragm or parasternal NME as a disease marker in *mdx* mice and most likely in DMD.

TTI is a critical measure of respiratory muscle function and risk of fatigue (Bellemare & Grassino, 1982; Dassios & Dimitriou, 2019; Miller & Mayer, 2021). Our study revealed that the *mdx* mouse differs substantively in TTI progression up to 16 months of age compared with progressive disease in human DMD. Surprisingly, TTI remains unchanged across all age groups and conditions in *mdx* mice. Moreover, no change in inspiratory duty cycle or respiratory muscle effort was observed. During increased respiratory demand, wild‐type mice increase total respiratory cycle time to maintain low TTI values, which was strikingly evident during sustained occlusion with increased expiratory pause between powerful inspiratory efforts. A similar response was observed in *mdx* mice.

Hahn et al. (2009) measured *P*_0.1_ (occlusion pressure at 0.1 s) and breathing patterns in 46 healthy males and 46 age‐matched participants with DMD. TTI was significantly higher in DMD patients compared to age‐matched controls (Hahn et al., 2009). Reduced MIP was a large contributor to the increase in TTI, consistent with the work of Mulreany and colleagues who investigated TTI in a smaller cohort of children with various neuromuscular diseases including children with DMD (Hahn et al., 2009; Mulreany et al., 2003). In patients with DMD, TTI increases beyond the threshold for fatigue and increases further, later in the disease, concurrent with the elaboration of major dysfunction (Khirani et al., 2014). TTI has emerged as a crucial measure in assessing respiratory function in DMD, offering a more comprehensive evaluation than single time point measurements of maximal inspiratory and expiratory pressures (Fauroux et al., 2015; Khirani et al., 2014). TTI has value due to its capacity to capture the complex interplay of factors affecting respiratory muscle function, including increased respiratory load, decreased muscle strength or a combination of both. It emerges as a robust predictor of respiratory failure in NMD patients, demonstrating strong inverse correlations with inspiratory vital capacity and peak inspiratory pressure (Miller & Mayer, 2021). Our study revealed that TTI is remarkably well maintained in *mdx* mice up to 16 months of age, notwithstanding that several other hallmark features of dystropathology are evident (e.g. diaphragm remodelling, weakness and impaired EMG activity), again revealing a remarkably well‐preserved respiratory capacity in *mdx* mice over much of the natural history of the disease.

As such, the *mdx* mouse model of DMD presents a conundrum for researchers interested in respiratory performance. There is profound diaphragm morbidity presenting early in the disease. Indeed, inspiratory pressure‐generating capacity (O'Halloran et al., 2023) and pressure–time relationship (Figure 9) are significantly impaired in 1‐month‐old *mdx* mice, consistent with diaphragm weakness, but respiratory performance is thereafter compensated and remarkably stable up to 16 months of age, with limited evidence of ventilatory insufficiency, and stability in several key clinically relevant indices of neuromuscular and neuromechanical performance. This raises concerns about the relevance of the model for human DMD or at least suggests that integrated respiratory assessments in *mdx* mice are best restricted to ≥16 months of age to end‐stage disease. This poses logistical challenges highlighting that alternative animal models are needed.

Other mouse models may better recapitulate the clinical respiratory phenotype (Delaney & O'Halloran, 2024; Mhandire et al., 2022). Among these, the dystrophin–utrophin null (*mdx*/*utrn* ^−/−^) mouse model of DMD appears promising (Hernández Rodríguez et al., 2025), showing early manifestation of ventilatory insufficiency by 6 weeks of age due to rapid progressive neuromuscular disease with profound kyphosis leading to premature death in early adulthood. Further characterization of respiratory function in the latter and related models of DMD is necessary to fully capture disease trajectory, which also needs to be fully established in people with DMD (Trucco & O'Halloran, 2025). Bridging this translational gap requires harmonization of electrophysiological biomarkers, manometry and imaging to provide a reliable and predictable platform for interventional trials.

### 4.1 Limitations

It can be difficult to reliably compare EMG signals between muscles and animals. To facilitate comparisons, we adopted a systematic approach to EMG recording and analysis. Electrode placement was standardised a priori (O'Halloran et al., 2023). All signals were processed in the same fashion (amplification, filtering, integration). Integrated EMG data are presented with absolute units to allow for faithful comparisons between muscles and between groups, that is, data are not obscured by presenting relative changes as percentage changes or normalised to a reference value. The principal conclusion we have drawn from EMG analyses is that there is lower activation in *mdx* mice particularly during high demand behaviours (O'Halloran et al., 2023), which we have also seen in follow‐up studies (Maxwell et al., 2024, 2025; Slyne et al., 2025). The intramuscular electrode measures multi‐unit activity, which is phasic with inspiration. The altered EMG signal in *mdx* mice reflects lower amplitude motor units. We quantified the composite signal using the standard approach of measuring the amplitude of the rectified integrated signal (the outcome is the same if area under the curve of the integral is assessed) with further analyses as described in the current manuscript. Loss of large amplitude units is discernible from our recordings sampled at 20 kHz. However, spike sorting of motor units would be required to fully characterise these changes in *mdx* muscles. We acknowledge that assessments were made under general anaesthesia, which depresses motor drive and hence the complexity of EMG signals. We acknowledge too technical limitations of EMG recordings per se that include electrode impedance, the relatively small area of muscle sampled in each recording, and the potential for artifact due to current spread from adjacent muscles, which may especially apply to intercostal muscle recordings.

We have inferred peak respiratory system performance from assessments of protracted tracheal occlusions. We determined that peak respiratory EMG activities coincide with the nadir pressure achieved during sustained occlusion, held until task failure. We have not observed greater values for inspiratory pressure or respiratory EMG across any other behaviours under our experimental conditions. Therefore, our assessment is of the maximum provoked values that can be achieved in the experimental preparation, which we have referred to as peak responses. Notably, to achieve peak pressures it was necessary to extend tracheal occlusion trials to >25 s as the early response in mice is characterised by rapid breathing and enhanced yet considerably sub‐maximal pressures. A secondary response in late occlusion was associated with respiratory slowing and pronounced peak pressures that could only be maintained for short periods (∼10–15 efforts) before task failure. Because animals are anaesthetised, we acknowledge that there is likely some attenuation of the ‘true’ maximum values that might be achieved in the conscious state.

We acknowledge a wealth of prior studies in rodents which report sub‐maximal transdiaphragmatic pressure changes during airway obstruction compared with maximum transdiaphragmatic pressure achieved during bilateral phrenic stimulation (Greising et al., 2016; for review see Sieck & Fogarty, 2025). Notably, in these studies, the duration of occlusions is shorter than that employed in our study (e.g. ∼15 s in total for 10 escalating efforts). This differs from the approach adopted in the current study (up to 30–40 s), which demonstrably achieves peak inspiratory pressures, since occlusions were held to task failure. It is most likely that we achieved maximal neural drive via the respiratory motor system and maximal neuromechanical action under the experimental conditions.

We acknowledge that transdiaphragmatic pressure is the gold standard assessment of diaphragm function in situ. However, our research question was broader than assessment of diaphragm performance in *mdx* mice compared to wild‐type mice. Rather, we were curious to determine respiratory capacity per se, which relates to the composite action of multiple inspiratory muscles, not just the diaphragm. Therefore, we assessed peak inspiratory pressure generated by the concerted action of all inspiratory muscles. Given that diaphragm force is severely curtailed in *mdx* mice, it is given that transdiaphragmatic pressure is lower in *mdx* mice, curiously even at 4 months of age (when peak inspiratory pressure is enhanced owing to compensation; see O'Halloran et al., 2023). As such, one could easily be misled by assessments of transdiaphragmatic pressure alone in *mdx* mice, given that the parameter is often taken as a proxy for peak inspiratory performance (Burns, Lucking et al., 2019. As demonstrated in our previous study (O'Halloran et al., 2023) there is a greater contribution by accessory muscles in *mdx* mice compared to wild‐type mice to peak inspiratory pressure generation.

### 4.2 Conclusion

Despite profound dystropathology of the major muscles of breathing, the *mdx* mouse shows remarkable compensation of respiratory system performance across much of the natural history of disease. We assessed several clinically relevant measures of respiratory neuromuscular and neuromechanical performance and found that these indices were generally equivalent in wild‐type and *mdx* mice. As such, the *mdx* mouse, which is the most widely studied model of DMD, offers logistical challenges for the assessment of ventilatory insufficiency, a key hallmark of human DMD. Interventional studies focussed on breathing in *mdx* mice are likely best restricted to ≥16 months of age to end‐stage disease (unless the focus relates to muscle form and function). It is necessary to implement and characterise models that better mirror the progressive respiratory decline in human DMD, albeit that the natural history of respiratory compromise in DMD also remains to be fully characterised, revealing an opportunity for bi‐directional translational research (Trucco & O'Halloran, 2025). Prioritizing EMG‐integrated monitoring frameworks could enable earlier detection of respiratory decline and personalised therapeutic interventions in DMD management.

## AUTHOR CONTRIBUTIONS

Michael N. Maxwell: experimental design; data and statistical analysis and interpretation of data; preparation of figures and drafting of the manuscript. Christopher G. Wilson: EMG analyses, interpretation of data; drafting, editing and critical review of the manuscript. Mai K. Elmallah: study design; interpretation of data; editing and critical review of the manuscript; Federica Trucco: study design; interpretation of data; editing and critical review of the manuscript. Ken D. O'Halloran: acquisition of funding; experimental design; supervision; acquisition of EMG and pressure data; interpretation of data; drafting, editing and critical review of the manuscript. All authors have approved the final version of the manuscript and agree to be accountable for all aspects of the work in ensuring that questions related to the accuracy or integrity of any part of the work are appropriately investigated and resolved. All persons designated as authors qualify for authorship, and all those who qualify for authorship are listed.

## CONFLICT OF INTEREST

Michael N. Maxwell, Christopher G. Wilson, Mai K. Elmallah and Ken D. O'Halloran declare that they have no competing interests. Federica Trucco reports participation in Scientific Advisory Boards for Roche UK and Italy and teaching initiatives for Biogen, Avexis, Roche and BREAS.

## Data Availability

All data are presented in the manuscript. Individual data points are shown for most data sets. Beyond the thorough descriptions provided in the text, all pertinent information on analyses will be openly shared upon reasonable requests.
